# White-tailed deer (*Odocoileus virginianus*) fawn survival and the influence of landscape characteristics on fawn predation risk in the Southern Appalachian Mountains, USA

**DOI:** 10.1371/journal.pone.0288449

**Published:** 2023-08-31

**Authors:** Adam C. Edge, Jacalyn P. Rosenberger, Cheyenne J. Yates, Andrew R. Little, Charlie H. Killmaster, Kristina L. Johannsen, David A. Osborn, John C. Kilgo, Karl V. Miller, Gino J. D’Angelo

**Affiliations:** 1 Daniel B. Warnell School of Forestry and Natural Resources, University of Georgia, Athens, Georgia, United States of America; 2 Virginia Department of Wildlife Resources, Marion, Virginia, United States of America; 3 School of Natural Resources, University of Nebraska-Lincoln, Lincoln, Nebraska, United States of America; 4 Wildlife Resource Division, Game Management Section, Georgia Department of Natural Resources, Social Circle, Georgia, United States of America; 5 USDA Forest Service, Southern Research Station, New Ellenton, South Carolina, United States of America; Cheetah Conservation Fund, Namibia University of Science and Technology, NAMIBIA

## Abstract

In the Southern Appalachian region of the United States, harvest data has indicated the occurrence of low deer densities while exposing a trend of declining white-tailed deer (*Odocoileus virginianus*) populations over the past several decades in northern Georgia. A triumvirate of increasing fawn predator populations reside in the Southern Appalachian Mountains including coyotes (*Canis latrans*), black bears (*Ursus americanus*) and bobcats (*Lynx rufus*). This region is also characterized by a homogenous landscape composed of mature forests and sparse understory vegetation, likely lacking adequate cover to offer fawns refugia from predators. Our objectives were to estimate survival and cause-specific mortality rates of fawns while assessing a possible link between mortality risk, intrinsic fawn characteristics (i.e., birth mass, Julian birth date, sibling status), and landscape features within fawn usage areas. During 2018–2020, we radio-collared 71 fawns within the Chattahoochee National Forest of northern Georgia, USA and monitored survival to 12 weeks of age. We observed low fawn survival (cumulative = 0.157, 95% CI = 0.091–0.273; vaginal implant transmitter = 0.196, 95% CI = 0.096–0.403) with predation as the leading cause of all known mortalities (45 of 55 mortalities; 82%) due primarily to coyotes (*n* = 22), black bears (*n* = 12), and bobcats (*n* = 7). Relationships between landscape features and fawn predation risk were minimal with only one informative covariate. Increasing amounts of early successional land cover within fawn usage areas decreased fawn mortality risk within the first 20 days of life, but elevated mortality risk thereafter. All fawns with any amount of early successional land cover in their usage areas died of predation (*n* = 13) at various time intervals, suggesting limited areas of potential fawning cover may be targeted by predators. However, fawn predation risk seemed to be high regardless of landscape covariates due to the limited number of surviving fawns. Coyote-caused mortality occurred over a longer period at a consistently higher magnitude than all other forms of mortality, indicating possible delayed prey-switching behavior and coyote predation as an important factor of fawn survival. The low recruitment of fawns influenced by high predation rates and homogenous habitat conditions is likely the cause of deer population declines in the region.

## Introduction

White-tailed deer (*Odocoileus virginianus*) densities in the Southern Appalachians region are known to be low [1] with harvest data documented in northern Georgia further suggesting deer populations have been in decline over the past few decades [2]. Thus, deer population sustainability concerns in the mountainous region were brought to the forefront. During 1979–2015, the Georgia Department of Natural Resources (GDNR) recorded an 85% and 97% reduction in hunter harvest for male and female deer, respectively, on 8 Wildlife Management Areas (WMAs) within the Chattahoochee National Forest of northern Georgia [2]. A 68% decrease in hunter participation also occurred on these WMAs during 1979–2018 [3] contributing to the decrease in harvest. However, buck harvest success rates (i.e., bucks harvested /hunter/days available) still fell by 64% during this time, even when hunters of these WMAs have indicated they mainly target bucks with minimal interest in harvesting does [3]. The reduction of buck harvest success was a likely indicator of decreasing deer densities [4]. Restriction or elimination of antlerless harvest opportunities by GDNR to stabilize or reverse population declines have not been successful [3]. Decreasing deer densities may have contributed to improved herd condition as mean dressed weights and main antler beam sizes of yearling bucks increased by 21% and 92%, respectively, during the same time period [2]. Therefore, nutritionally related declines in herd health and fecundity over the last few decades are unlikely [5], and decreased fawn recruitment has been considered as a likely driver of population decline necessitating investigation within the region.

Deer populations throughout the southeastern United States (U.S.) generally are abundant [1]. However, recent localized declines in fawn recruitment have been attributed to the range expansion of coyotes (*Canis latrans*; [6–8]). Studies throughout the southeastern U.S. have recorded fawn survival in the range of 14–43% with the majority of mortalities resulting from coyote predation [8–12]. Furthermore, coyote removal efforts in the region have suggested coyotes contribute a potential source of additive mortality with evidence of improved fawn survival following their reduction on some sites [13–16] and a lack of compensatory response by other forms of fawn mortality during years of reduced coyote predation [15]. American black bears (*Ursus americanus*) have also been documented as a leading contributor to fawn mortality rates in some studies [17–19] with bobcats (*Lynx rufus*) affecting survival of fawns as well but to a lesser degree [10,18]. This triad of fawn predators have all become well-established in the Southern Appalachians in recent decades, changing the predator context on the landscape. Coyotes first arrived to northern Georgia in the 1980s and now have established breeding populations throughout the eastern U.S. [20]. Although current density estimates are lacking, coyote and bobcat numbers are purportedly increasing across most of the southeastern U.S. [6,21]. Black bears were considered rare in parts of the Southern Appalachians until the 1970s due to near extirpation at the beginning of the century [22]. Since then, bear populations in northern Georgia have grown rapidly at an estimated annual growth rate of 1.07–1.08 suggesting a 381% population increase during 1979–2012 [2].

Most deer populations in the southeastern U.S. are able to maintain stable or growing populations in the presence of multiple predators with adjusted antlerless harvest regulations [23–25]. Thus, variation in fawn survival rates and examples of reduced fawn recruitment suggest other environmental factors may interact with predation to facilitate population declines. Fawns generally select bed sites in denser vegetation to reduce predator detection while in the hider stage [26]. However, on sites in the southeastern U.S. recently colonized by coyotes, only weak or non-existent relationships have been described between fawn survival and understory vegetation structure at birth sites or fawn locations [8,15]. In the vicinity around fawn birth sites, Watine and Giuliano [11] reported fawn survival was negatively related to basal area and canopy cover of forest stands and positively related to shrub density. At the landscape level, both Rohm et al. [27] and Gulsby et al. [28] found the likelihood of coyote predation increased as edge density decreased and as habitat became more homogenous within areas of fawn use. Vreeland et al. [17] did not find a relationship between fawn survival and habitat patch diversity or edge density but concluded that fawn survival was higher in open agricultural areas compared to forested areas. A meta-analysis of North American white-tailed deer fawn survival studies concluded that mortalities due to predation were highest in contiguous forest areas and less in areas of agriculture and mixed forest/agriculture [29]. Therefore, while multiple levels of habitat likely influence fawn survival in synchronicity [11], landscape structure may be the most determinant scale.

The landscape of the Southern Appalachians embodies a large expanse of forested land cover with intermittent rural development. Contiguous forests with a primarily mature, closed-canopy structure typical of the mountainous region can negatively influence fawn survival [29]. A lack of recent forest disturbance through a history of fire-suppression [30] and declining timber harvest [31] has led to <1% of the land cover on the Chattahoochee National Forest (CNF) in northern Georgia being composed of early successional habitat or young forest, with 88% of forest stands ≥61 years old [2]. Under-representation of early seral stages is a common condition across the Southern Appalachian region, with natural resource agencies expressing concerns about the homogenous forest composition [32]. Forest-dwelling deer are primarily associated with disturbance, which creates intermixed canopy gaps and increased edge within contiguous forest [33]. Expansive closed-canopy forests have insufficient availability of understory cover needed by fawns as refugia from predators [26] and may lack nutritious forage needed by lactating females [34] increasing risk of maternal neglect [35]. Inadequate abundance and distribution of quality forage could force female deer to spend more time foraging in risky areas [36,37]. Furthermore, limited soft mast in the understory and midstory of mature closed-canopy forests could increase predation of fawns by omnivores such as coyotes and black bears [38,39] by restricting prey-switching behavior [40]. Anthropogenic developments can also influence predation risk of fawns [41] as human structures act either as a safeguard to fawns due to deterrence of predators from human activity [42] or as a predator attractant due to the availability of unnatural foods [43,44].

We investigated white-tailed deer fawn survival in the Southern Appalachian region of northern Georgia with the objectives of quantifying fawn survival trends, assessing the impact of predation and other forms of mortality on fawn recruitment, evaluating relationships between intrinsic fawn characteristics (i.e., birth mass, Julian birth date, and sibling status) and mortality risk, and assessing potential links between predation risk and landscape-level characteristics within fawn usage areas. We expected fawn survival rates to be in the lower range of rates found within the southeastern U.S. (14–43%) due to high levels of predation within homogenous, highly forested habitats of fawn usage areas. The combination of high predator densities and a landscape lacking habitat diversity can have detrimental effects on fawn survival [27,28,45]. We also predicted sources of fawn mortality to differ temporally as fawns age due to predator-specific ability and hunting strategy [46].

## Material and methods

### Study area

**Fig 1:**
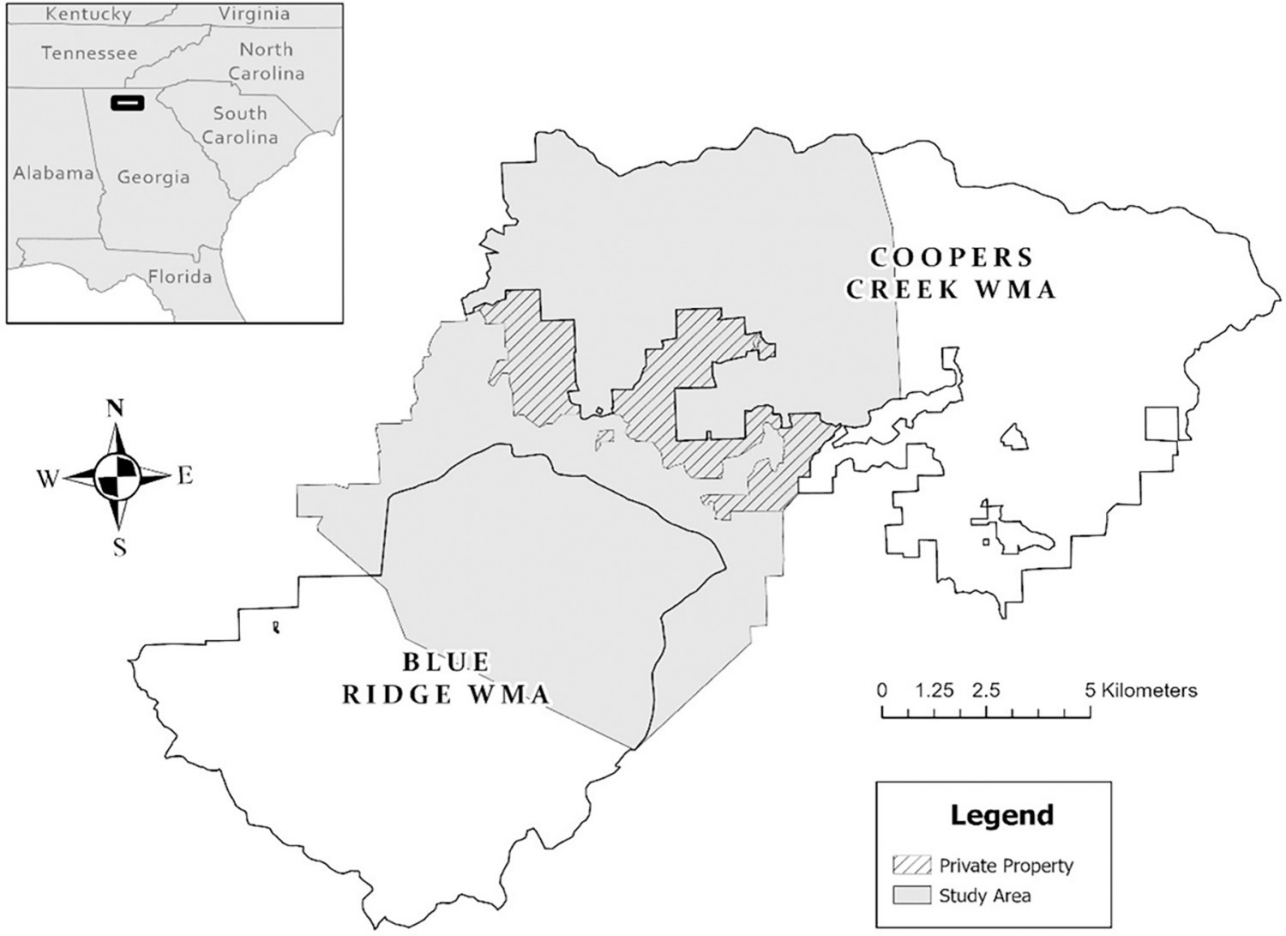
Study area included portions of Coopers Creek and Blue Ridge Wildlife Management Areas within the Chattahoochee National Forest and intermixed private land holdings in northern Georgia, USA.

We conducted our research within Fannin and Union counties in northern Georgia, USA on a 135–km^2^ study area composed of portions of the CNF (89%) and private land holdings (11%; Fig 1). Segments of the 85–km^2^ Blue Ridge and 121–km^2^ Coopers Creek WMAs, which are federally owned lands cooperatively administered with the GDNR for wildlife management, make up most of the CNF included in our study area. The total CNF consisted of 3038 km^2^ of public land heavily utilized for recreational activities such as hiking, camping, hunting, and fishing.

The study area was generally characterized by closed canopy, late successional forest with steep slopes and drainages at elevations of 450–1350 m. Average annual temperatures can vary from 6.3 to 19.9°C with annual rainfall and snowfall averaging 142 cm and 10.2 cm, respectively. Tree species at elevations below 915 m included white oak (*Quercus alba*), black oak (*Q*. *velutina*), chestnut oak (*Q*. *montana*), hickories (*Carya* spp.), eastern white pine (*Pinus strobus*), and Virginia pine (*P*. *virginiana*; [47]). Trees most common in mid-to-upper elevations were yellow poplar (*Liriodendron tulipifera*), black birch (*Betula lenta*), red maple (*Acer rubrum*), northern red oak (*Q*. *rubra*), chestnut oak, American basswood (*Tilia americana*), and various montane yellow pines (*Pinus* spp.; [47]). North-facing slopes were dominated by upland hardwoods while south-facing slopes had a mixed hardwood-pine structure [47]. On more mesic sites and along drainages or river bottoms, rosebay rhododendron (*Rhododendron maximum*) dominated the understory and midstory forming dense patches whereas mountain laurel (*Kalmia latifolia*) was common on more xeric sites [48]. Other species commonly found in the understory and/or midstory included American holly (*Ilex opaca*), greenbriers (*Smilax* spp.), and various species of *Vaccinium* spp. [47]. Early successional land cover and young forest were greatly lacking, potentially leading to limited soft mast production. Wildlife openings were planted and maintained on Blue Ridge and Coopers Creek WMAs to provide additional wildlife forage but covered <1% of our study area (0.1 km^2^). Private lands offered open areas in the form of mowed lawns, old fields, and pasture lands used for harvesting hay and cattle grazing. Forested areas of private land were frequently parceled into small, developed lots with cabin structures.

Forest management activities have changed over time in the Chattahoochee-Oconee National Forests in Georgia. From the early 1960s through the early 1990s, timber harvest often exceeded a cut volume of 283,168 m^3^ of solid wood per year (USDA Forest Service, S1 Table). Timber harvest declined to an average cut volume around 5,663 m^3^ per year in the 2000s as pressure from forest advocacy groups elevated and more restrictive federal regulations slowed the process, as illustrated by Hoberg [49]. Since 2005, timber harvest has trended upward again, reaching 112,953 m^3^ in 2017, but still does not approach quantities of earlier decades. Prescribed burning on the forest incrementally increased from 29 km^2^ to 67 km^2^ burned in 2003 and 2016, respectively (USDA Forest Service, S2 Table). When considering the 3,038 km^2^ National Forest with closed-canopy conditions, the overall habitat improvement impact of fire has likely been limited at a large scale.

Deer harvest began to decline most drastically in the 2000s [2], which may be attributed to changing habitat conditions within a maturing forest structure and growing predator numbers. Current deer density estimates in the CNF are 1.9–3.9 deer/km^2^ [2] as compared to 7.3–9.3 deer/km^2^ in 1993 on Blue Ridge and Cooper’s Creek WMAs [50]. Deer hunts have been actively managed on the WMAs since the first managed hunt in 1940 on the Blue Ridge Game Management Area (now Blue Ridge WMA; [51]). Currently, deer hunts occur during September–January with regulations prohibiting female harvest and firearm hunts restricted to specific days on these WMAs.

### Adult female capture and handling

During January to mid-April of 2018–2020, we captured female deer ≥1.5 years of age throughout the study area. We used whole kernel corn (*Zea mays*) to lure deer for capture via rocket-nets, drop-nets, Clover traps [52], and tranquilizer darts using a 2.0-cc combination of butorphanol, azaperone, and medetomidine (BAM, Wildlife Pharmaceuticals, Windsor, Colorado) intramuscularly to chemically immobilize deer [53]. We applied Vetericyn ophthalmic gel (Innovacyn, Inc., Rialto, California) to maintain lubrication of eyes, blindfolded deer, and monitored vitals throughout the handling process. We fitted deer with GPS radio-collars (1-fix every 4 hours; Vectronic Aerospace Gmbh, Berlin, Germany) and numbered ear tags (Cattlesoft Inc., College Station, Texas, USA), measured morphological characteristics, and noted body condition. Assuming all females were pregnant, we also inserted vaginal implant transmitters (VITs) to facilitate subsequent capture of fawns. Most females had VITs with a Neolink ultra-high frequency external sensor function (Vectronic Aerospace Gmbh, Berlin, Germany) that sent an email message via satellite through the corresponding collar when the VIT was expelled. We inserted very high frequency (VHF) VITs (Advanced Telemetry Systems, Inc., Isanti, Minnesota, USA) without Neolink capabilities into some deer as these devices were smaller in diameter and could be more easily received by females with a constricted vaginal canal (i.e., adults resisting sedation). Yearling females (1.5 years of age) were non-receptive to both VIT types possibly due to their nulliparous physiology and smaller body size. We still collared yearlings to observe potential localization related to parturition and assist fawn capture. We antagonized BAM with an intramuscular injection including 4.0 cc of atipamezole plus 0.5 cc of naltrexone (Wildlife Pharmaceuticals, Windsor, Colorado; [53]). We monitored deer until they recovered and released them at the site of capture. Capture and handling of deer was approved by the University of Georgia Institutional Animal Care and Use Committee through Animal Use Protocol #: A2016 11-015-Y1-A0.

### Fawn capture and handling

We monitored collared females from 15 May until the last female gave birth each season. We used radio-telemetry to check VITs on 8-hour intervals as the Neolink feature was found to be unreliable in the mountainous landscape. Once a VIT was expelled at birth, we waited for ≥2 hours to allow the doe time to groom, feed, and bond with her young [54]. Females may move their fawns rather quickly from the birth sites, so expedited searches facilitated capture. When a birth site was indicated (i.e., expelled VIT), we checked the vicinity for fawns and grid-searched an approximate 100-m radius around the site. If no fawn was found, we would return to search again the next day. In instances of defective VITs (e.g., loss of VHF beacon) and of females that were not implanted with VITs (e.g., yearlings, adults resisting full sedation), we checked GPS fixes daily for a clustering of locations, which often was evidence of a birth site. Upon possible birth site detection, we searched for fawns in the vicinity of the location clusters. We used digital thermal scopes (American Technology Network Corp., San Francisco, California, USA) in lower light conditions to aid in locating fawns. We supplemented fawn capture efforts with random searches along roads, pastures, wildlife openings, and forests during peak fawning periods in 2019 and 2020. Fawns captured via clustering of GPS fixes and random searches are hereby referred to as opportunistic captures.

Once found, we blindfolded the fawn with cloth, weighed it in a pillowcase with a digital hanging scale, applied small ear tags (Cattlesoft Inc., College Station, Texas, USA), fitted the expandable breakaway VHF collar (4-hr mortality delay; Vectronic Aerospace Gmbh, Berlin, Germany), and measured hoof characteristics. We estimated ages of fawns caught opportunistically based on hoof growth (i.e., measuring from hairline to growth-ring line on inward side of front hoof), hoof development (i.e., soft, hard, gray color, white color, and gelatinous material), umbilicus condition (i.e., bloody, moist, present and dry, absent and scabbed, and absence of scab), and behavior (i.e., remained bedded, little struggle, struggled vigorously, and fled during approach; [55,56]). We assumed capture mass of fawns ≤1 day old equaled birth mass. When we found twins, we processed and collared both as independent samples as predation has been found to influence sibling fawns independently due to spatial separation [57]. Handling times averaged 14.1 min ± 0.87 (SE) and fawns were released at their site of capture.

### Fawn monitoring and fate determination

We monitored fawn survival from birth until either death or 12 weeks of age. During the first 4 weeks of life, which is considered the interval of greatest mortality [8,9,27], we monitored fawns with telemetry at 8-hour intervals. Thereafter, we monitored fawns once per day until 12 weeks old. During telemetry checks, we also triangulated one location every day for the first 4 weeks of life. We estimated locations using Universal Transverse Mercator coordinates with the maximum likelihood estimator in program LOAS 4.0 (Ecological Software Solutions, Sacramento, CA, USA) and only accepted locations with error ellipses ≤2 ha.

We evaluated mortalities immediately after they were discovered to reduce the possibility of scavenging, thereby aiding in determination of cause of death [58]. We assigned causes of mortality after careful examination of field evidence at the recovery site using Alt and Eckert [46] as a guide, genetic testing methods described by Kilgo et al. [9], and necropsy by the Southeastern Cooperative Wildlife Disease Study (SCWDS, University of Georgia, Athens, Georgia, USA). We photographed and analyzed all field evidence that included presence of subcutaneous hemorrhaging (i.e., confirmed sign of predation), carcass caching, carcass burial, tracks, scat, hair, drag marks, claw marks, and kill-site conditions. Species-specific predator determinations were further reinforced with the use of DNA analysis (Wildlife Genetics International Inc., Nelson, British Columbia, Canada) from possible predator saliva sampled from fawn carcasses and/or fawn collars using cotton swabs. We took multiple DNA swabs during each fawn mortality investigation. We preserved carcasses found whole with no signs of predatory attack in cool storage and sent them to the SCWDS lab to determine cause of death.

We designated a probable cause of death with the categories of predation (e.g., coyote, black bear, bobcat, unknown), natural causes (e.g., failure to thrive, disease/birth defects), and human-caused (e.g., mower, vehicle collision). Failure to thrive included instances of emaciation, possible starvation resulting from maternal neglect, and adverse effects due to environmental conditions. Analysis of predator DNA swabs can return results matching dog/wolf genetics due to their introgression in the genome of southeastern coyotes [59]. Kilgo et al. [9] confirmed through further genotyping analysis that all dog/wolf samples (*n* = 7) in their study were predominantly coyote in genetic composition. Therefore, we considered any dog/wolf DNA samples as coyote. We acknowledge the possibility of predators masking other underlying susceptibilities or causes of death [60]. However, our intensive monitoring schedule was devised to provide confidence in our ability to arrive at mortality sites before scavenging took place, allowing us to accurately assess non-predation mortalities. Fawn survival studies using similar monitoring periods did not observe a single scavenging event on fawn carcasses that died of non-predator related causes and concluded a low likelihood of bias using DNA-based predator identification [8,9]. Therefore, we remained consistent with our predation determination if DNA evidence was present [28], even if only a collar was recovered at a mortality site since coyotes and black bears are known to consume fawn carcasses entirely [46]. Bobcats rarely consume entire carcasses, but instances can occur [61]. We determined a collar as prematurely dropped if there was no carcass found, no DNA evidence of predation present, and collar condition or position indicated possible drop-off. Marking-induced mortality was unlikely as all fawns were handled similarly to other studies and previous research has indicated this as a low risk for white-tailed deer fawns [62–64].

### Habitat characterization

To assess the effects of habitat characteristics on fawn predation risk, we developed fawn usage areas using triangulated fawn locations. We used a subset of fawns for this analysis that included only those that succumbed to predation or survived to the end of the 12-week monitoring period (*n* = 54). This subset excluded non-predation fawn mortalities and fawns with failed collars. We could not develop individual fawn home ranges due to fawn predation events taking place early in life before a sufficient number of locations could be collected. Therefore, we used methodology similar to Vreeland et al. [17] and Gulsby et al. [28] to develop buffered areas around fawn capture locations for comparison. We used R software (version 4.1.3) package ‘adehabitatHR’ [65] to create 95% Minimum Convex Polygons (MCPs) for fawns with ≥15 locations within their first 4 weeks of life ($\bar{x}$ = 25 locations) resulting in a mean MCP area of 14.3 ± 2.7 (SE) ha (*n* = 25). Therefore, we created a 14.3-ha circular buffer around the capture site of each fawn in the subset to represent a standardized usage area using ArcGIS Pro 2.9.0 (Environmental Systems Research Institute, Inc., Redlands, CA, USA). The same software was used for all subsequent geographic information systems operations.

To measure habitat composition within fawn usage areas, we obtained 30-m grid cell resolution layers for elevation (Digital Elevation Model, United States Geological Survey 2016) and land cover (National Land Cover Database 2019). We reclassified land cover types to include biologically relevant land cover classes: deciduous forest (e.g., hardwoods), evergreen forest (e.g., pines), mixed forest (e.g., hardwood/pine), early successional vegetation (e.g., shrub/scrub, herbaceous, wildlife openings), and pasture/grass (e.g., cattle grazing pastures and lawns). We reassigned developed land cover classes to the nearest neighboring class because we represented anthropogenic developments (e.g., human structures and roads) by feature class layers. We obtained feature layers for paved and unpaved roads (USDA Forest Service 2016) and waterways (National Hydrology Dataset, U.S. Geological Survey 1999) to calculate the length of linear features (m) within fawn usage areas. We added any roads missing from the feature layers within the study area using satellite imagery. Waterways included both primary and secondary water sources (i.e., rivers and streams). Telemetry data indicated fawns <4 weeks of age crossed waterways, so they were not a complete barrier to movement. We developed a point feature layer that marked human structures using satellite imagery to calculate the density of human structures within fawn usage areas. Lastly, we delineated an understory evergreen layer by geo-locating mountain laurel and rosebay rhododendron patches and using image classification to train winter satellite imagery for areas that were not sampled. We tested the accuracy of the layer we created by ground-truthing the existence of mountain laurel or rosebay rhododendron at randomly selected points indicated by the satellite imagery training. Mountain laurel and rosebay rhododendron were of interest due to their common dominance in the understory and midstory and their potential to provide a limited source of cover for fawns.

We clipped each raster (i.e., elevation, land cover, understory evergreen cover) to the circular fawn usage areas. We imported the elevation rasters of each fawn usage area into R software to calculate mean terrain ruggedness index (TRI; [66]) accounting for topographic variation in the mountainous landscape. We uploaded the land cover and understory evergreen rasters of each fawn usage area into FRAGSTATS (UMass Landscape Ecology Lab) to quantify landscape and class level land cover covariates (Table 1). We clipped waterways and roads within each fawn usage area to measure the total length (m) of each linear feature.

**Table 1:** Description of landscape, class, and feature covariates measured within white-tailed deer fawn usage areas used to model the effects of predation risk on fawns in northern Georgia, USA, 2018–2020.

Variable name	Metric type	Definition
Shannon’s diversity index	Landscape	Measure of relative patch diversity
$\bar{x}$ patch fractal dimension	Landscape	Measure of shape complexity
$\bar{x}$ terrain roughness index	Landscape	Measure of landscape topography
Total forest cover	Class	Total area (ha) of all forest type patches
Evergreen understory cover	Class	Total area (ha) evergreen cover in understory
Deciduous forest cover	Class	Total area (ha) deciduous forest
Evergreen forest cover	Class	Total area (ha) evergreen forest
Mixed forest cover	Class	Total area (ha) mixed (deciduous + evergreen) forest
Early successional cover	Class	Total area (ha) early successional vegetation
Pasture/grass cover	Class	Total area (ha) pasture or lawn
Building density	Feature	Sum of human structures/ area
Paved road	Feature	Length (m) of paved roads
Unpaved road	Feature	Length (m) of unpaved roads
Waterways	Feature	Length (m) of rivers and streams combined
Forest edge	Feature	Length (m) of boundaries shared between forest patches and non-forest patches

We chose FRAGSTATS metrics to characterize habitat heterogeneity based primarily on covariates previously determined important to fawn survival in the literature including Shannon’s diversity index (SHDI; [28]), mean patch fractal dimensions [28], amount of forest cover [27,29], edge length [27,28], and evergreen understory cover (Table 1). Interspersion juxtaposition index (IJI), which is a measure of patch adjacency, was also found to be important [28] but can only be calculated when measured areas have >2 land cover types. Only 62% of our fawn usage areas contained >2 land cover types, so we did not include the metric. Habitat edges have been deemed important to fawn survival as an indicator of habitat complexity affecting evasion of predators by fawns [27,28], possible use as travel corridors for predators [67], or areas of high prey densities targeted by predators [68]. Gulsby et al. [28] concluded that high-contrast edges contributed the most to fawn survival such as those between forest and fields. Therefore, we measured forest-field edge within fawn usage areas as the combined length (m) of boundaries shared by forest type patches (i.e., deciduous, mixed, evergreen) and open type patches (i.e., early successional, pasture/grass). We defined edges between land cover classes as one pixel (30 m x 30 m) in size. Total area (ha) of land cover types per fawn usage areas was also calculated.

### Statistical analysis

We used R software (version 4.1.3) with the package ‘survival’ [69] to estimate fawn survival rates and assess the effects of covariates on survival. We developed Kaplan-Meier (KM) survival curves [70] through 12 weeks of age generalized for the case of staggered entry [71] using an age-based time scale [72]. We right-censored fawns that exited the monitoring period early due to premature collar drop or collar failure. Opportunistically captured fawns may have been several days old at capture, leading to potential overestimation of survival and biased ecological conclusions due to inclusion of left-truncated data [60,73,74]. Therefore, we modeled survival curves for the cumulative fawn data (i.e., VIT and opportunistic captures) and VIT-captured fawn data independently. Subsequently, we assessed competing mortality risks using the Non-Parametric Cumulative Incidence Function Estimator function ‘NPCIFE.R’ [75] as an extension of the KM estimator to develop survival curves of competing mortality risks (e.g., coyotes, black bears, bobcats, naturally occurring) for both datasets. This approach allowed us to account for the mutually exclusive nature of mortality events and to evaluate the contribution of each cause of death independent of other causes by censoring non-referenced mortality sources [76]. We were also able to observe temporal trends describing the timing of cause-specific mortality events in association with fawn age.

We used Cox proportional-hazards (CPH) regression models [77] to determine mortality risk associated with intrinsic fawn characteristics for the separate fawn data sets (i.e., cumulative and VIT). Previous simulation studies have recommended at least 10 events (i.e., mortalities) per predictor variable (EPV) are needed when using CPH models to avoid over-fitting [78,79]. Vittinghoff and McCulloch [80] determined this rule to be slightly over-conservative as an EPV of 5–9 performed similarly to an EPV of 10–16. The use of binary predictors has also been found to contribute to increased bias with lower EPV and sample size [80,81]. Therefore, we considered these constraints when developing our *a priori* candidate models. We assessed covariates indicated by the literature to assess possible importance to fawn survival, particularly those that could be impacted by management or have an effect on subsequent model structure, including birth mass [18], Julian birth date [9], and sibling status [8]. Sex has been found to be an important predictor of fawn survival in some studies as well [19], but we were unable to add the covariate to our model set due to limitations described above. Due to the inclusion of fawns captured >1 day after birth in our analysis, we developed a linear growth curve of age versus mass to calculate fawn mass gain per day as fawn growth has been found to follow a linear trend [56]. The fawn mass gain per day based on the linear equation was 0.12 kg/day which was consistent with the 0.13 kg/day growth rate found by Zwank et al. [82] in Louisiana. Therefore, we backdated the mass measurements of older fawns by 0.12 kg/day to day 1 birth mass. We based Julian birth dates on known (i.e., VIT captures) and estimated birth dates (i.e., opportunistic captures). We only included sibling status in the model set using VIT fawn data as the presence of a twin could not be verified for opportunistic captures.

We also applied CPH models to assess the potential impact of habitat characteristics on fawn predation risk within the 12-week monitoring period based on our subset of developed fawn usage areas (*n* = 54). We combined opportunistically (*n* = 26) and VIT-captured (*n* = 28) fawns in this analysis due to the limitations of a reduced sample size when separating the data. We acknowledge the concerns of including left-truncated data in this analysis, but low sample sizes can influence model interpretation as well [80,81]. Our subsampled data also omitted fawns dying of natural causes which Chitwood et al. [74] found to be the mortality factor most influenced by inclusion of opportunistic fawn data. We developed candidate models *a priori* using a hypothesis-based modeling approach to address 3 specific hypotheses:

*Hypothesis 1*: *Landscape heterogeneity*–Fawn predation risk will decrease in fawn usage areas with more heterogeneous habitat. Included covariates: SHDI, mean patch fractal dimension, mean TRI, understory evergreen cover, total forest cover, forest edge.
*Hypothesis 2*: *Land cover*–Fawn predation risk will increase in fawn usage areas containing more forested land cover and decrease with more open land cover. Included covariates: deciduous forest cover, mixed forest cover, evergreen forest cover, early successional cover, pasture/grass cover.
*Hypothesis 3*: *Fawn predator evasion*–Fawn predation risk will decrease in fawn usage areas containing more human structures and linear road features because human presence offers protection by predator deterrence. Included covariates: building density, paved roads, unpaved roads, waterways, forest edge.

We tested the proportional hazards assumption for each model using the ‘cox.zph’ function with a significance of *p* < 0.05 indicating disproportional hazards. If the mortality hazard associated with a covariate was found to violate this assumption and be time-varying, we used the ‘survSplit’ function to stratify the mortality risk effect of the covariate into time intervals based on beta inflection points [83]. We used Pearson’s correlation coefficient to test for autocorrelation among all covariate pairs. Pairs with coefficients of *r* > |0.7| were not included in the same model set. We scaled covariates by their standard deviations (SD) for interpretability. Competing models were chosen using the Akaike Information Criterion (AIC) weighted approach corrected for small sample sizes (AICc) with plausible models ≤2.0 AICc units from the top approximating model [84]. Akaike weights (*w*_*i*_) were used to evaluate competing model strength, and log likelihoods were assessed to verify informative covariates [85].

## Results

We captured and collared 59 females ≥1.5 years old (2018 = 12, 2019 = 23, 2020 = 24) with 14 of those deer recaptured in succeeding years (2019 = 2, 2020 = 12) for a total of 73 capture events (2018 = 12, 2019 = 25, 2020 = 36). Therefore, recaptured females may have received more than one VIT throughout the study. Including recaptures, we deployed 55 VITs (2018 = 9, 2019 = 19, 2020 = 27). Only 21 of 55 VITs (38%) resulted in the successful capture of ≥1 fawn. VITs not resulting in captures (*n* = 34) were due to transmitter malfunction (*n* = 16), failure to locate fawns of dams with seemingly functional VITs (*n* = 9), no indication of VIT drop (*n* = 3), death of female before parturition (*n* = 3), early VIT expulsion (*n* = 2), and a landowner denied permission to search on private land (*n* = 1). We captured 70 fawns (39 M, 31 F) over 3 years with 11 fawns (5 M, 6 F) caught in 2018, 21 fawns (14 M, 7 F) caught in 2019, and 38 fawns (20 M, 18 F) caught in 2020. We included an additional fawn in 2020 of unknown sex that was confirmed to be predated before our arrival, due to fresh carcass remains near the birth site consistent with predation, yielding a total sample of 71 fawns. We captured 30 fawns from VITs (2018 = 8, 2019 = 5, 2020 = 17) and 41 fawns opportunistically using random searches (2018 = 1, 2019 = 12, 2020 = 20) and observations of clustered female collar fixes (2018 = 2, 2019 = 4, 2020 = 2). One fawn captured using localization of collar fixes came from a yearling that was not receptive of a VIT.

We estimated a conservative birth rate of 1.43 fawns/female from 30 VIT-captured fawns known to be born from 21 collared females. The average birth mass of fawns caught ≤1 day of parturition (*n* = 26) was 2.40 ± 0.10 (SE) kg for males (*n* = 13) and 2.30 ± 0.10 (SE) kg for females (*n* = 13). Standardized birth masses of all fawns differed by year (*F*_2,66_ = 8.570, *p* < 0.001) with year 2 birth masses (1.99 ± 0.09 [SE] kg) being less than year 3 (2.56 ± 0.10 [SE] kg; *p* < 0.001). The cumulative mean date of birth was June 23 (i.e., known and estimated birth dates) with no difference among years (*F*_*2*,*68*_ = 1.459, *p* = 0.240). All captured fawns were born between May 27 and July 17. The average estimated age of fawns captured opportunistically and by VIT was 4.1 and 1.3 days old, respectively. We were unable to locate some VIT-related fawns until the second or third day after birth (*n* = 8), but we knew the fawns to be associated to a VIT due to their close proximity to the birth site and dam GPS locations.

Fawn monitoring concluded with a total of 55 known mortalities (77%), 9 known survivors (13%), 4 premature collar drops (6%), and 3 collar failures (4%). Of the 55 mortalities, 45 were due to predation (82%) and 10 were due to other natural causes (18%; Table 2). We observed no human-caused mortalities. We received genetic results for 40 of 45 fawn predation events with 19 being coyote, 10 black bear, 6 bobcat, 2 dog/wolf, 1 with both coyote and wild pig (*Sus scrofa*), 1 with both coyote and bobcat, and 1 with both black bear and house cat (*Felis silvestris catus*). We included dog/wolf samples with coyote samples. The mortality including coyote and wild pig DNA was designated coyote predation as wild pigs have not been confirmed to be a predator of fawns in any southeastern fawn survival study [8,9,10,19,86], and any speculative wild pig predation is expected to be very low [87]. Therefore, we deemed coyotes responsible for 22 total fawn mortalities. We designated the mortality event with both bobcat and coyote DNA evidence as a bobcat kill, assuming it more likely the fawn was killed by a bobcat and stolen/scavenged by a coyote than vice versa [9], yielding 7 total bobcat predation events. We designated the predation event including both black bear and house cat DNA as black bear predation due to the fawn skull being found crushed with multiple puncture wounds consistent with a bear kill strike, yielding 11 black bear mortalities. Only 1 of 40 predation events where DNA evidence was found contained DNA from two verified predator species (i.e., coyote/bobcat) supporting our species-specific designations. Of the 5 predation events with inconclusive DNA evidence, we were able to confidently designate a specific predator for one of the mortalities based on field evidence. The mortality site contained bone fragments, a bloody collar, nearby fresh bear scat, and a bear was spotted fleeing the site upon arrival. Therefore, we designated cause of death as bear predation, yielding 12 total bear predation events. We were unable to determine the predator responsible for the other 4 mortalities because field evidence was consistent with both coyote and bear. However, we were able to confirm predation as the cause of death due to evidence of hemorrhaging from punctures on carcasses or carcass remains and presence of blood. We designated cause of death for these 4 mortalities as unknown predator. Of the 10 mortalities due to other natural causes, necropsy revealed causes of death were due to failure to thrive (n = 9) and disease/birth defects (n = 1). Failure to thrive determination was characterized by emaciation, exposure to unfavorable weather conditions possibly causing hypothermia, possible maternal neglect resulting in starvation, or other underlying morbidity factors undetectable by necropsy. One fawn had a heart deformity that likely led to cardiac fibrosis as the cause of death.

**Table 2:** Mortality causes of collared white-tailed deer fawns to 12 weeks of age in northern Georgia, USA, 2018–2020. Data represented 55 mortality occurrences from the monitoring of 71 total fawns (2018 = 11, 2019 = 21, 2020 = 39).

Cause of Mortality	2018		2019		2020		Total	
	__________		__________		__________		__________	
	n	%	n	%	n	%	n	%
**Predation:**								
Coyote^a^	4	50.0	5	29.4	13	43.3	22	40.0
Black bear	2	25.0	4	23.5	6	20.0	12	21.8
Bobcat	1	12.5	1	5.9	5	16.7	7	12.7
Unknown^b^	0		2	11.8	2	6.7	4	7.3
**Natural causes:**								
Failure to thrive^c^	1	12.5	4	23.5	4	13.3	9	16.4
Disease/birth defects^d^	0	0.0	1	5.9	0	0.0	1	1.8
**Human-caused:**								
Mower	0	0.0	0	0.0	0	0.0	0	0.0
Vehicle-collision	0	0.0	0	0.0	0	0.0	0	0.0
**Total**	**8**	** **	**17**	** **	**30**	** **	**55**	** **

^a^Includes 2 mortalities with dog/wolf genetic results from 2019 assumed to be coyote.

^b^Combination of field evidence consistent with multiple predators and no usable DNA samples.

^c^Includes carcasses found whole with possible causes of death due to emaciation, maternal neglect, unfavorable weather conditions.

^d^Mortality caused by cardiac fibrosis due to heart deformity.

When assessing the fate of VIT-captured fawns only we observed a similarly high number of mortalities at 24 (80%) with 6 known survivors (20%). Most mortalities were caused by predation (n = 22, 92%) followed by a low number of naturally occurring deaths (*n* = 2, 8%). Of the 22 predation events, evidence designated 10 to coyotes (45%), 8 to black bears (36%), 3 to bobcats (14%), and 1 to an unknown predator (5%).

**Fig 2:**
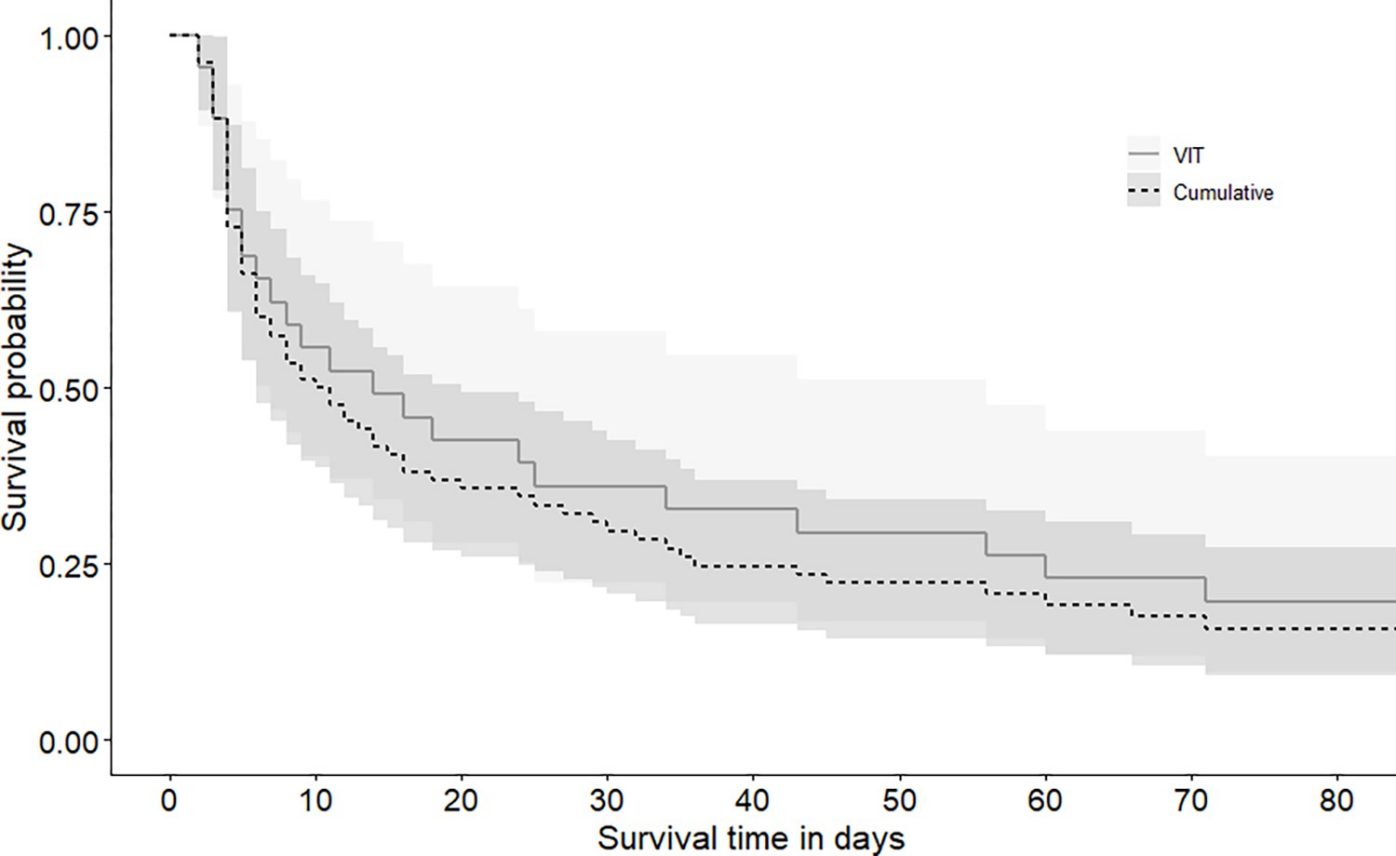
Daily Kaplan-Meier estimate for cumulative survival (*n* = 71) and vaginal implant transmitter (VIT) survival data (*n* = 30) collected from radio-collared white-tailed deer fawns to 84 days (12 weeks) of life in northern Georgia, USA, 2018–2020. Each ‘step’ indicates a mortality event while corresponding shaded areas depict 95% confidence intervals.

The Kaplan-Meier fawn survival estimate to 12 weeks of age was lower for cumulative fawn data than VIT-only fawn data at 0.157 (95% CI = 0.091–0.273) and 0.196 (95% CI = 0.096–0.403), respectively (Fig 2). Upon visual inspection, the two survival curves were initially converged until day 5 when cumulative survival dropped slightly below VIT survival. Most mortalities occurred early in life for both scenarios as survivorship was 0.500 (95% CI = 0.386–0.648) by day 10 for cumulative survival and 0.491 (95% CI = 0.341–0.708) by day 14 for VIT survival. Neither cumulative (*X*^*2*^ = 1.1, *p* = 0.6) or VIT (*X*^*2*^ = 2.3, *p* = 0.3) fawn survival statistically differed across years.

**Fig 3:**
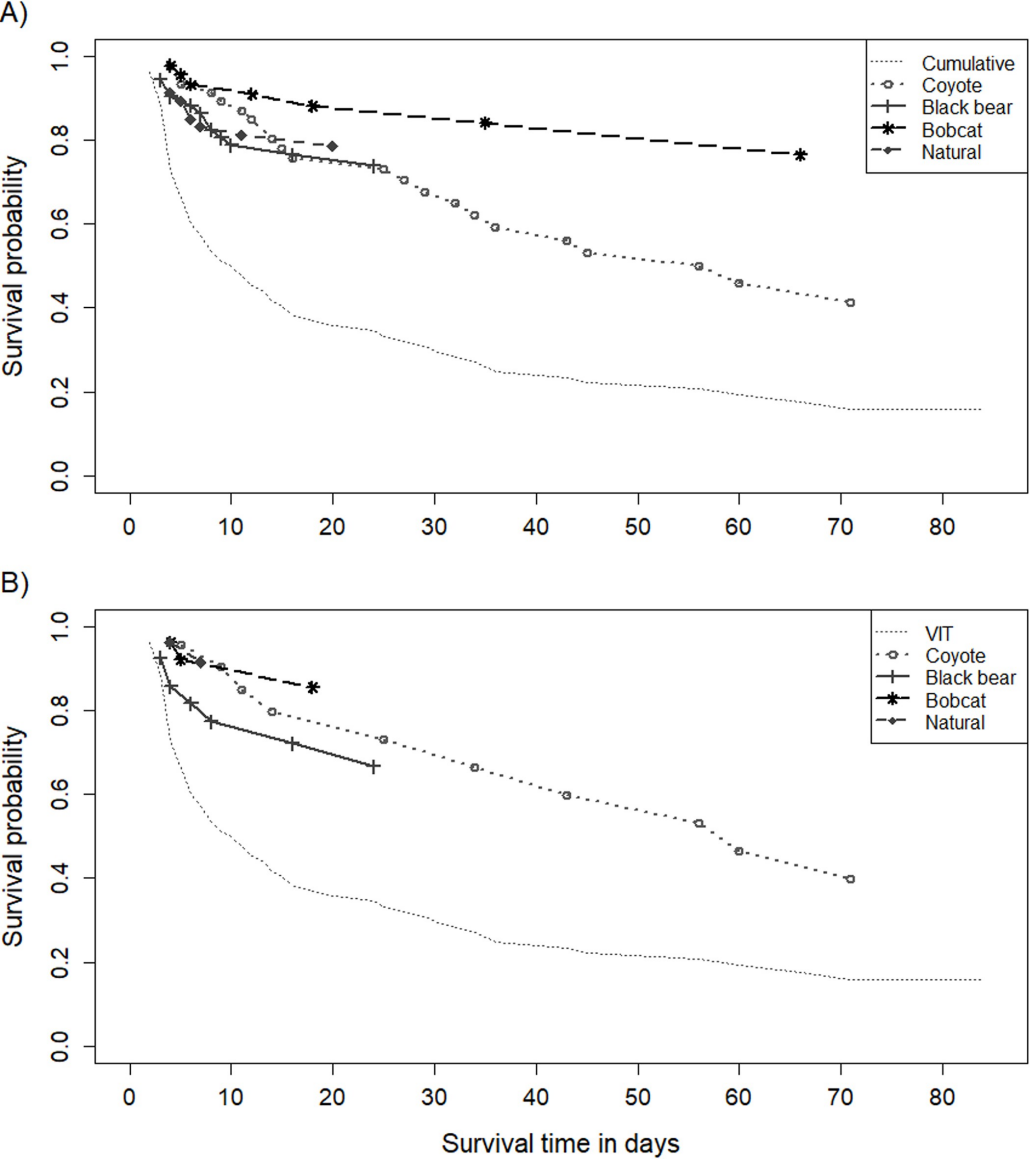
Contribution of specific causes of mortality (e.g., coyotes, black bears, bobcats, naturally occurring) to estimated Kaplan-Meier white-tailed deer fawn survival rates of cumulative (*n* = 71; A) and vaginal implant transmitter (VIT; *n* = 30; B) fawn data in northern Georgia, USA, 2018–2020. All other sources of mortality were censored in each survival curve associated with a specific mortality cause. Symbols indicate the occurrence of mortality events within the first 84 days (12 weeks) of life for radio-collared fawns.

Different sources of mortality had variable effects on total fawn mortality. When assessing the cumulative dataset, the impact of bobcats on fawn survival was minimal compared to other predators and was sporadic over time, resulting in a fawn survival rate of 0.764 to 12 weeks old (*n* events = 7) if bobcats were the only source of mortality (Fig 3A). The last bobcat predation event occurred at 66 days of age. Black bear predation only occurred early in the fawn monitoring period concluding with a fawn survival rate of 0.739 (*n* events = 12) due solely to bears with the last bear predation event taking place at 24 days of age. The fawn survival rate due to coyotes as the only mortality source was similar to bears during the first 4-week period of highest fawn susceptibility (0.704). However, coyote predation continued at a consistent rate until the end of the monitoring period with a final fawn survival rate of 0.414 to 12 weeks old (*n* events = 22). The oldest fawn mortality due to coyote predation was 71 days old. Other naturally caused mortalities (i.e., failure to thrive, disease/birth defects) only took place during the first 20 days of life contributing to a fawn survival rate of 0.785. Thus, coyotes were the primary factor influencing overall fawn survival in our study due to the total number of fawns predated by coyotes and their consistent contribution to mortality hazard over an extended period of time.

The VIT dataset revealed similar trends when comparing competing mortality sources. Fawn survival was high at 0.855 (*n* events = 3) if bobcat predation was the only mortality source (Fig 3B). The last bobcat mortality occurred at 18 days of fawn age, which is a younger age than the cumulative data suggested bobcats were able to successfully ambush fawns. Black bears had a higher mortality effect with a fawn survival rate of 0.664 (*n* events = 8) if bear predation was the only cause of mortality. However, the last black bear predation event still occurred at 24 days of age with VIT fawns. Coyotes again contributed to the lowest fawn survival rate of 0.398 (*n* events = 10) as a sole mortality source due to the extended and consistent predation risk they pose. The last coyote mortality event involved a 71 day old fawn. The effect of other natural moralities was very minimal with fawn survival being 0.915 (*n* events = 2) without predation. The last naturally caused mortality occurred at 7 days old for VIT fawns.

When analyzing mortality risk associated with fawn survival to 12 weeks of age and intrinsic fawn covariates, birth mass was the most informative predictor in the top models for both the cumulative and VIT model sets accounting for Akaike weights of *w*_*i*_ = 0.63 and *w*_*i*_ = 0.50, respectively (Table 3). However, mortality risk interpretation was different for each. The hazard ratio associated with birth mass in the cumulative model set (β = -0.08, SE = 0.24, HR = 0.92, 95% CI = 0.578–1.466) indicated an 8% reduction in mortality risk with every 1-kg increase in fawn mass, whereas the birth mass hazard ratio in the VIT model set (β = 0.05, SE = 0.5, HR = 1.05, 95% CI = 0.396–2.797) indicated a 5% increase in mortality risk with every 1-kg increase in fawn mass. The average birth mass of fawns known to survive to the end of the monitoring period and fawns succumbing to mortality was 2.59 ± 0.23 (SE) kg (*n* = 9) and 2.33 ± 0.07 (SE) kg (*n* = 55), respectively, for the cumulative dataset. Surviving fawns and fawns succumbing to mortality weighed 2.25 ± 0.21 (SE) kg (*n* = 6) and 2.31 ± 0.07 (SE) kg (*n* = 23), respectively, for the VIT dataset. We did not find sibling status as a covariate effecting mortality risk to be important, further verifying our initial collaring of both twins.

**Table 3:** Hazard ratio models assessing the influence of intrinsic fawn covariates on the mortality risk of radio-collared white-tailed deer fawns to 12 weeks of age in northern Georgia, USA 2018–2020. Model sets are differentiated by use of cumulative fawn data (*n* = 71) and data including only fawns captured via vaginal implant transmitter (VIT; *n* = 30).

Model	*K*	AIC_c_	ΔAIC_c_	*ω*_i_	log L
Cumulative					
Mass^a^	1	371.65	0.00	0.63	-184.79
Global^b^	2	372.75	1.10	0.37	-184.25
Null	0	384.39	12.74	0.00	-192.19
JDOB^c^	1	385.85	14.20	0.00	-191.89
VIT					
Mass	1	131.24	0.00	0.50	-64.52
Mass + JDOB	2	133.47	2.23	0.17	-64.44
Mass + sibling^d^	2	133.62	2.38	0.15	-64.51
Null	0	135.24	4.00	0.07	-67.62
Global	3	135.57	4.33	0.06	-64.15
Sibling	1	137.35	6.11	0.02	-67.58
JDOB	1	137.41	6.17	0.02	-67.62
JDOB + sibling	2	139.64	8.40	0.01	-67.53

^a^Mass = standardized mass at birth.

^b^Global = combination of all covariates used in each model set.

^c^JDOB = Julian date of birth.

^d^Sibling = presence of a twin, only included with VIT model set.

Several covariates were autocorrelated and not included in the same model assessing effects of landscape covariates on predation risk. SHDI was correlated with forest edge (*r* = 0.84) and total forest cover (*r* = -0.76), while forest edge was also correlated with total forest cover (*r* = -0.93). The early successional cover covariate did not meet the proportional hazards assumption of CPH models, meaning the covariate effect on mortality risk varied over time. Therefore, we stratified the covariate by time intervals associated with disproportionate changes in predation risk.

*Hypothesis 1*: *Landscape heterogeneity*–Habitat complexity (mean patch fractal dimensions) was the only covariate in the most supported model (*w*_*i*_ = 0.23; Table 4) and the hazard ratio (β = -0.28, SE = 0.15, HR = 0.76, 95% CI = 0.562–1.023) revealed a 24% decrease in predation risk for every 1-SD-unit (0.02) increase in habitat complexity within fawn usage areas. The next competitive model included SHDI (*w*_*i*_ = 0.14), and every 1-SD-unit (0.27) increase in habitat diversity increased predation risk by 26% (β = 0.23, SE = 0.14, HR = 1.26, 95% CI = 0.950–1.660). However, the null model was also competitive (*w*_*i*_ = 0.12) indicating predation risk was likely not associated with covariates in this model set.
*Hypothesis 2*: *Land cover–*The top model and only competing model included early successional cover (*w*_*i*_ = 0.43; Table 4). To account for violation of the proportional hazards assumption, we stratified the hazard effect of the early successional cover covariate into 3 time intervals (strata 1: 1–6 days of age, strata 2: 7–20 days of age, strata 3: 21–84 days of age). Fawns were exposed to variable levels of predation risk at different ages depending on the amount of early successional cover in their usage areas. For every 1-SD-unit (0.45 ha) increase in early successional cover in fawn usage areas fawn mortality risk decreased by 45% during strata 1 (β = -0.59, SE = 0.85, HR = 0.55, 95% CI = 0.106–2.910) and 10% during strata 2 (β = -0.10, SE = 0.51, HR = 0.90, 95% CI = 0.335–2.432). However, for every 1-SD-unit (0.45 ha) increase in early successional cover during strata 3 (β = 1.67, SE = 0.53, HR = 5.31, 95% CI = 1.882–14.976) the probability of fawn predation increased 431%, although the confidence interval was large. Of the 13 fawns in the subset that had some amount of early successional cover within their usage areas, 2 died within strata 1, 5 died within strata 2, and 6 died within strata 3, all of predation.
*Hypothesis 3*: *Fawn predator evasion–*Unpaved roads was the only covariate in the top model (*w*_*i*_ = 0.25) with the hazard ratio indicating for every 1-SD-unit (242.71 m) increase in unpaved road length fawn predation risk decreased by 23% (β = -0.27, SE = 0.16, HR = 0.77, 95% CI = 0.563–1.598; Table 4). However, the next competitive model was the null model (*w*_*i*_ = 0.18) implying covariates in this model set were likely uninformative.

**Table 4:** Hazard ratio models assessing the influence of landscape covariates on the predation risk of radio-collared white-tailed deer fawns to 12 weeks of age in northern Georgia, USA 2018–2020. Model sets were developed using a hypothesis-based approach associated with landscape heterogeneity (Hypothesis 1), land cover (Hypothesis 2), and fawn predator evasion (Hypothesis 3). Models used a subset of fawns (*n* = 54) including only those that survived or succumbed to predation.

Model	*K*	AIC_c_	ΔAIC_c_	*ω*_i_	log L
Hypothesis 1					
Complexity^a^	1	291.28	0.00	0.23	-144.59
Diversity^b^	1	292.23	0.95	0.14	-145.07
Complexity + diversity	2	292.6	1.32	0.12	-144.16
Null	0	292.64	1.36	0.12	-146.32
Complexity + understory^c^	2	293.32	2.04	0.08	-144.51
Complexity + TRI^d^	2	293.4	2.12	0.08	-144.56
Diversity + TRI	2	294.32	3.04	0.05	-145.02
TRI	1	294.68	3.40	0.04	-146.30
Understory	1	294.69	3.41	0.04	-146.30
Diversity + complexity + TRI	3	294.8	3.52	0.04	-144.11
Diversity + complexity	3	294.89	3.61	0.04	-144.15
+ understory					
Global^e^	4	297.21	5.93	0.01	-144.11
Hypothesis 2					
Early successional: strata^f^	3	300.08	0.00	0.43	-146.75
Early successional: strata	4	302.48	2.40	0.13	-146.74
+ pasture/grass					
Deciduous forest	1	302.99	2.91	0.10	-150.45
Null	0	303.04	2.96	0.10	-151.52
Mixed forest	1	304.54	4.46	0.05	-151.22
Early successional: strata	6	304.83	4.75	0.04	-145.31
+ Mixed forest + deciduous					
forest + evergreen forest					
Deciduous forest + mixed forest	2	304.91	4.83	0.04	-150.31
Evergreen forest	1	305.06	4.98	0.04	-151.48
Pasture/grass	1	305.13	5.05	0.03	-151.52
Mixed forest + evergreen forest	2	306.14	6.06	0.02	-150.93
Deciduous forest + mixed forest	3	307.02	6.94	0.01	-150.22
+ evergreen forest					
Global	7	307.15	7.07	0.01	-145.06
Hypothesis 3					
Gravel road	1	291.93	0.00	0.25	-144.92
Null	0	292.63	0.70	0.18	-146.32
Paved road	1	292.99	1.06	0.15	-145.45
Gravel road + paved road	3	293.99	2.05	0.09	-143.70
+ waterway					
Edge	1	294.47	2.53	0.07	-146.19
Buildings^g^	1	294.60	2.67	0.07	-146.26
Buildings + paved road	4	294.85	2.91	0.06	-142.93
+ gravel road + waterway					
Gravel road + waterway + edge	3	295.28	3.34	0.05	-144.35
Gravel road + paved road + edge	3	295.29	3.35	0.05	-144.35
Buildings + edge	2	295.81	3.88	0.04	-145.76
Global	5	297.39	5.45	0.02	-142.93

^a^Complexity = mean patch fractal dimension.

^b^Diversity = Shannon’s diversity index.

^c^Understory = evergreen understory including mountain laurel and rosebay rhododendron.

^d^TRI = terrain roughness index.

^e^Global = combination of all covariates used in each model set.

^f^Early successional: Strata = early successional land cover stratified by 3 time intervals.

^g^Buildings = number of human structures.

## Discussion

Our study provided an evaluation of white-tailed deer fawn survival in a region of the Southern Appalachians with closed-canopy, homogenous forests and a triad of fawn predators. We observed cumulative and VIT-only fawn survival rates to 12 weeks of age at 0.157 and 0.196, respectively, which are among the lowest recorded survival rates in North America (0.141–0.900; [29]). Potentially, the fawn survival rates in our study could have been lower due to events where we could not find fawns at VIT-drop locations (n = 9), as fawns may be preyed upon soon after birth before opportunity for capture [19]. We confirmed one fawn mortality prior to capture during our study. The use of opportunistically captured fawns when assessing survival can upwardly bias survival rates often leading to ecological misinterpretations [60,73,74]. However, our cumulative fawn survival rate (0.157, *n* = 71), which incorporated opportunistic fawns, was lower than the VIT-only rate (0.196, *n* = 30), so overestimation was not a concern. Therefore, our data suggests a low sample size of non-truncated data could also potentially bias survival estimates. Gilbert et al. [73] concluded that a balanced combination of truncated and non-truncated data could be used to estimate survival as long as certain standards were met. We suggest further assessment is needed to weigh the use of combined data types versus lower sample sizes of non-truncated data. Our results emphasize the value of estimating survival rates separately by fawn capture type for comparison and transparency of ecological conclusions.

Fawn survival studies completed in the southeastern U.S. have found predation to be the main cause of death for fawns, with coyotes acting as the primary source of mortality: 21–83% [64], 37–80% [9], 52% [10], 55% [8], 66% [7], 74% [11]. The only exception occurred in another 3-predator system in Louisiana where black bear predation was the greatest source of fawn mortality at 33% [19]. Many southeastern U.S. fawn studies attribute recent localized declines in fawn recruitment to high levels of predation [6–8,10], which is evidenced in our study as well. Fawn mortality risk was previously lower in the Southern Appalachians with fawn recruitment rates estimated at 0.82–0.83 fawns/female in 1983 [88] compared to our current data that suggests a low fawn recruitment rate of 0.22–0.28 fawns/female (birth rate × fawn survival), likely in response to burgeoning predator populations. A similar scenario has occurred on the Savannah River Site in South Carolina where recruitment rates ranged from 0.81–1.27 fawns/adult female during 1965–1990 and declined to 0.21–0.55 fawns/adult female during 1999–2006 after the establishment of coyotes to the region [6]. Overall, fawn survival was higher on most other southeastern U.S. study sites with greater reported reproductive rates as well at 1.70–1.75 fawns/female [6,25,89]. A study in the Appalachian Mountains of eastern Kentucky recorded a lower birth rate of 1.36 fawns/female, but the fawn mortality rate was low as well (34%) allowing for sustainable deer populations [12]. Our study reported a slightly higher birth rate than eastern Kentucky at 1.43 fawns/female, but the profoundly high mortality rate of fawns compromise recruitment and population sustainability.

Reproductive productivity of Southern Appalachian deer populations has been historically marginal in relation to surrounding regions due to lower soil productivity [90] and the density-independent influence of acorn (*Quercus* spp.) availability likely limiting reproductive potential [91–93]. However, reproductive rates have remained stable over the past several decades on average as an adult fetal rate of 1.47 was recorded by Wentworth et al. [91] in our study region during 1983–1988, comparable to the conservative birth rate we observed (1.43 fawns/female). Southern Appalachian forests are nutritionally limited during the winter months, so deer are heavily reliant on energy stored from acorn consumption the preceding fall [91,94]. However, acorn production varies from year to year which can cause fluctuations in deer body condition [92,94]. Prior studies in the region found deer weights and kidney fat indices were greatest in the fall and continuing into winter following a good acorn crop [94], and ensuing yearling female reproductive rates were higher as well [91]. Following poor acorn crops, deer condition status declined [91,92,94] which can reduce ovulation [91], delay fetal growth [95], decrease mass at birth [95], and diminish milk production by the dam for nursing [35]. Furthermore, a study in the Central Appalachians found that acorn abundance was also related to annual fawn survival with mortality risk increasing after a fall of poor acorn production, due to maternal condition factors [96].

Acorn crop indices varied in the northern Georgia mountains during our study based on roadside surveys (2017 = good, 2018 = poor, 2019 = fair; GDNR, S1 Fig). We found fawns had significantly less birth mass in 2019 than in 2020 which coincided with the acorn availability of the previous falls and was potentially linked to maternal acorn consumption during gestation [95,96]. However, we would expect to find a larger difference in fawn birth mass following years of ‘good’ verses ‘poor’ acorn crops for this relationship to hold true, but we did not observe this. We also did not detect annual variation in fawn survival as low, variable sample sizes restricted interpretation. Cumulative data results did indicate that increased fawn mass at birth was a potential factor leading to decreased mortality risk as has been reported in many cervids [18,97,98], with all surviving fawns having a birth mass almost 11% more at birth on average than all fawns succumbing to mortality. However, results were in contradiction with our non-truncated dataset which indicated the opposite and made biological interpretation difficult to comprehend. Regardless, hazard effect sizes relating birth mass to fawn mortality risk were small while the influence of other intrinsic fawn covariates was negligible, potentially indicating the predominance of top-down predatory effects over bottom-up nutrition factors. Nonetheless, acorn mast should still be considered a potential factor in annual variation of fawn condition and survival as has been observed in studies of longer duration [91,92,96]. Although in our study region, harvest data has also suggested a long-term trend of improving population condition regardless of annual fluctuations [2]. The infrequent years of limited reproduction and potentially increased fawn susceptibility due to reliance on a density-independent factor can make deer populations in the Southern Appalachians more vulnerable to increasing predator abundances [91,96,99]. As evidence, fawn recruitment is now 4-times lower following the change in predator composition and abundance on the landscape than it was in the 1980s [88] when deer were still influenced by the same density-independent nutritional constraints [91].

We also assessed the influence of landscape-scale habitat characteristics on fawn susceptibility to mortality, finding mostly weak relationships. In contrast to previous research indicating visual concealment has little effect on fawn predation risk during the first 2–3 weeks of life in the southeastern U.S. [8,15], we found that early successional cover was initially beneficial to fawns by reducing mortality risk. At this time, fawns are in the hider stage [100], so early successional ground cover was likely important for concealment from predators in our system. However, at older ages, increasing amounts of early successional cover within fawn usage areas drastically increased fawn predation risk. Fawns become increasingly mobile as they age and begin to spend more time following their mothers [100]. This progression potentially increases fawn visibility as they expand their movements through other cover types with less visual obstruction at ground level. Thus, the limited availability of early successional cover on the landscape could be creating an ecological trap where predators may continually target the limited areas of perceived fawning cover [101] and successfully capture fawns at older ages. Only 24% of fawns in this analysis had any amount of early successional cover in their usage areas, and they all died of predation (*n* = 13) within 60 days of age.

The lack of relationships between fawn survival and landscape characteristics was likely due to limited sample size and the overall low number of surviving fawns with which to compare predation risks. Ultimately, it seemed fawns on our study site were at high risk of predation regardless of landscape characteristics. Low sample size may have also led to inflation of hazard ratios for strata 3 of the early successional land cover covariate, so effect size should be interpreted with caution. Autocorrelations among our landscape covariates also limited our assessment as a testament to the homogenous structure of our study environment. We additionally acknowledge the limitations of using circular buffers around fawn capture locations to represent fawn usage areas. Our buffers were developed based on fawn locations during the first 4 weeks of life [28], so spatial utilization of older fawns could have expanded beyond our buffers as fawns became more mobile with age. Conversely, fawns dying at young ages may have never fully utilized their calculated usage areas. However, we deemed this the best methodology to account for our high level of fawn mortalities before a sufficient number of locations could be collected while enabling standardized measures of the landscape within the vicinity of all fawns regardless of fate.

Although we were only able to detect one noteworthy relationship between predation risk and landscape characteristics, observations of temporal patterns related to competing mortality sources were more insightful. Both data types (i.e., cumulative, VIT) revealed similar conclusions, with predation by black bears and coyotes occurring frequently and at similar rates during the early weeks of fawn life. However, coyote predation continued through 10 weeks of age, while no black bear predation events occurred after fawns were 4 weeks old. Black bear predation of elk (*Cervus canadensis*) and caribou (*Rangifer tarandus*) calves similarly decreased as calves grew older [97,102]. Unlike coyotes, bears likely lack the endurance to capture increasingly mobile fawns [102], whereas the ambush strategy of bobcats allows continued effectiveness regardless of prey mobility [103]. However, research has suggested bobcats are constrained by interference from coyotes in open areas and homogenous habitats like our study area [104], and white-tailed deer are not a major component of bobcat diets in areas where they are sympatric with coyotes [10]. Thus, bobcat predation events occurred more sporadically over time and at a lower rate than other predators. Natural mortalities were similarly low, only occurring early in fawn life. Although, we acknowledge that abandonment or malnourishment can make fawns more susceptible to predation as a non-proximate cause of death [60]. For instance, we documented one fawn that ultimately died of emaciation and potential abandonment that was making loud, continual vocalizations similar to observations of Chitwood et al. [105]. These vocalizations could potentially attract predators and mask natural causes of mortality early in life. However, Chitwood et al [105] and our field crew found all carcasses from observed vocalizing fawns without signs of predation and were able to correctly designate those mortalities as naturally caused.

Several studies have indicated that coyote predation of fawns declines significantly after fawns reach about 4 weeks of age with some coyote related mortality events tapering out beyond this period [8,9,10]. However, our study found the risk of coyote predation continued at near the same rate after this 4-week period, while risk of death by other agents of mortality ceased or substantially reduced. Therefore, fawn survival in our study region may be subjected to additive mortality pressure from coyotes, as several southeastern studies have suggested [9,13,14,16] or directly evaluated [15], due to the magnitude and longevity of coyote contribution to fawn mortality. The extent of coyote predation risk may also explain why increased amounts of early successional cover amplified fawn predation risk beyond 3 weeks of age as coyotes were still actively targeting fawns and continued to catch them. Coyote predation accounted for a known 67% (*n* = 4) of fawn mortalities that occurred after 21 days of age (strata 3) when early successional cover was present in fawn usage areas, although sample size was low. An additional 17% (*n* = 1) of related fawn mortalities were attributed to an unknown predator with evidence consistent with possible coyote predation. Ward et al. [40] and Jensen [106] recorded a shift in coyote diet composition in the later summer months with increased consumption of fruits. Some studies have suggested that soft mast availability may buffer predation risk [9,40,106,107]. In our study, 41% (*n* = 9) of known fawn deaths due to coyotes occurred beyond 4 weeks of age suggesting a possible lag in prey-switching behavior due to a lack of early successional and young forest conditions which can provide abundant soft mast and alternative prey species [108,109].

Forest management practices that open the canopy have been shown to increase soft mast production and ground cover [109,110], while helping to create a balance of forest stands at different seral stages that can sustain patches of higher quality forage [111] and deer refugia throughout a landscape. Residual oak trees after a timber thinning can then increase acorn production due to crown release [112]. Populations of small mammals such as voles and mice have also been documented in higher numbers in areas following timber management than in undisturbed Appalachian forests [108]. All factors considered, site-specific conditions appear to act in concert to increase fawn mortality in northern Georgia, including fawn condition reliant on maternal condition factors [35], landscape composition [28], alternative food availability for predators [40], and predator densities [45]. In our study, low fawn recruitment due to high predation rates of young fawns coupled with marginal habitat conditions and relatively low fecundity are likely synergistically contributing to deer population declines.

## Conclusion

Limited fawn recruitment rates are seemingly the key contributor to deer population declines in northern Georgia and possibly throughout the entire Southern Appalachian region. These low rates are driven by high predation rates under a changing predator context, homogenous habitat conditions, and generally low deer productivity dependent on seasonally available acorn crops. In addition, a relative lack of active forest management (e.g., timber harvest, prescribed fire) on our Southern Appalachian study site has resulted in a landscape dominated by mature, closed-canopy forests lacking browse, soft mast production, and wildlife cover [111]. In synchronicity, these conditions of increasing predator abundances and mature forests are not conducive to fawn survival and, thus, growth of deer populations.

Options to reverse deer declines are limited. GDNR has restricted antlerless harvest on public land and reduced opportunity on private land in most of northern Georgia. While this management strategy can be beneficial, it likely will be insufficient to reverse population decline in a situation of heavy fawn predation pressure similar to the conclusions of Chitwood et al. [89]. Coyote control may produce variable results without long-term effectiveness across a large landscape due to their ability to quickly fill the void [113], although there is evidence of increased fawn survival when removal efforts are intensive and highly targeted on specific sites [13–16,86]. However, implementation may be controversial on national forest land from a public perception standpoint and logistically difficult. Increasing black bear harvest to maintain or reduce bear populations may have implications for improved fawn survival [114,115], but effectiveness has not yet been assessed in the southeastern states. Forest management efforts can enhance understory fawn refugia, habitat complexity, and early seral stage land coverage for improved predator escapability, while also providing alternative prey sources to potentially induce prey-switching behavior in predators and increase fawn survival. Timber harvest to improve winter browse will likely be ineffective as cold-season forage is more related to site characteristics in the Southern Appalachians, but opening the canopy can improve warm-season nutrition [92] important for lactation and maternal care during fawning [35]. Ultimately, it may take a combination of management strategies to improve fawn survival and promote deer population growth in northern Georgia.

## Supporting information

S1 Fig Oak mast survey index for northern Georgia mountains (1985–2021) provided by the Georgia Department of Natural Resources, Wildlife Resources Division, Game Management Section. (PDF)

S1 Table Annual timber cut volumes on the Chattahoochee-Oconee National Forests (1944–2017) provided by the USDA Forest Service. (PDF)

S2 Table Annual prescribed fire area burned on the Chattahoochee National Forest (2003–2016) provided by the USDA Forest Service. (PDF)

S3 Table Survival data for 71 collared white-tailed deer fawns monitored to 12 weeks of age in northern Georgia, USA (2018–2020). (PDF)

S4 Table Survival data for a subset of 54 collared white-tailed deer fawns that either survived to 12-weeks of age or succumbed to predation including landscape characteristics measured within fawn usage areas in northern Georgia, USA (2018–2020). (PDF)
