# Supplementary figures and images for: White-tailed deer (Odocoileus virginianus) fawn survival and the influence of landscape characteristics on fawn predation risk in the Southern Appalachian Mountains, USA
Source: PLoS One. 2023 Aug 31;18(8):e0288449. doi: 10.1371/journal.pone.0288449 (PMC10470973; doi:10.1371/journal.pone.0288449)

North Georgia Mountains Oak Mast Survey & Mortality Data 1985-2021

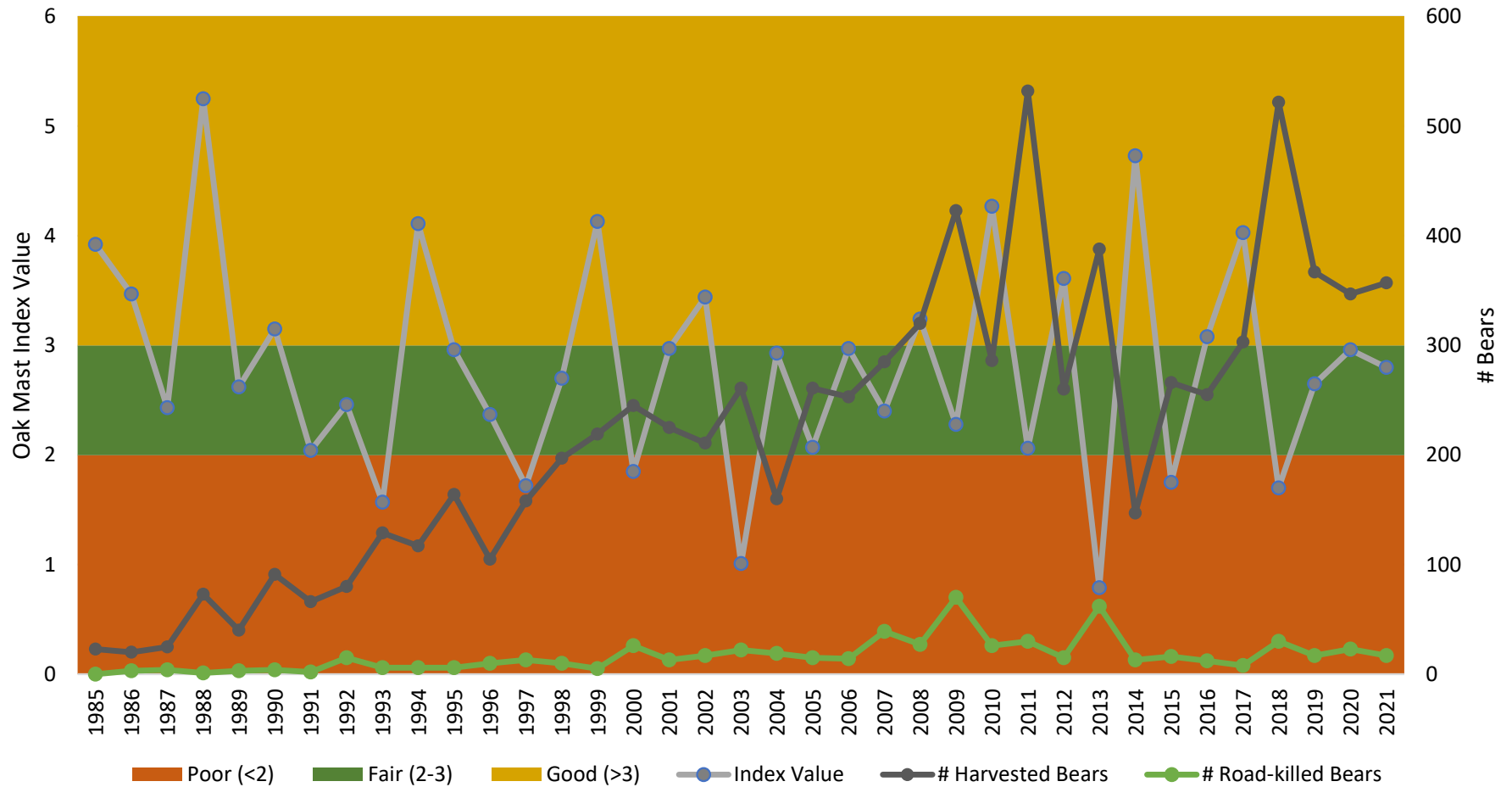

Supplement: S1 Fig — (PDF) [file pone.0288449.s001.pdf]
