# Supplementary material for: White-tailed deer (Odocoileus virginianus) fawn survival and the influence of landscape characteristics on fawn predation risk in the Southern Appalachian Mountains, USA
Source: PLoS One. 2023 Aug 31;18(8):e0288449. doi: 10.1371/journal.pone.0288449 (PMC10470973; doi:10.1371/journal.pone.0288449)
Supplement: S1 Table — (PDF) [file pone.0288449.s002.pdf]

Chattahoochee-Oconee National Forests Timber Harvest

Timber cut volumes – USDA Forest Service data

Fiscal Year	CCF (100 cubic feet)	m^3
1944	94,390	267,282
1945	83,568	236,638
1946	70,868	200,675
1947	107,316	303,885
1948	72,500	205,297
1949	67,202	190,295
1950	23,442	66,380
1951	93,040	263,460
1952	66,132	187,265
1953	44,152	125,024
1954	47,316	133,984
1955	57,510	162,850
1956	81,986	232,158
1957	61,426	173,939
1958	70,760	200,370
1959	79,006	223,720
1960	89,800	254,285
1961	85,274	241,469
1962	99,556	281,911
1963	97,326	275,596
1964	112,830	319,498
1965	105,316	298,221
1966	114,760	324,964
1967	108,884	308,325
1968	100,942	285,835
1969	115,982	328,424
1970	120,564	341,399
1971	120,898	342,344
1972	102,424	290,032
1973	103,118	291,997
1974	112,812	319,447
1975	116,860	330,910
1976	142,076	402,314
1977	103,972	294,415
1978	86,784	245,745

1979	127,730	361,690
1980	179,016	506,916
1981	94,526	267,667
1982	94,612	267,911
1983	109,882	311,151
1984	106,060	300,328
1985	91,734	259,761
1986	133,272	377,384
1987	141,600	400,966
1988	132,616	375,526
1989	137,878	390,426
1990	107,300	303,839
1991	104,232	295,152
1992	115,510	327,087
1993	108,892	308,347
1994	81,070	229,564
1995	56,694	160,539
1996	40,004	113,279
1997	54,178	153,415
1998	27,500	77,871
1999	18,527	52,463
2000	1,496	4,236
2001	2,012	5,697
2002	1,276	3,613
2003	2,958	8,376
2004	2,868	8,121
2005	6,528	18,485
2006	8,560	24,239
2007	11,842	33,533
2008	47,352	134,086
2009	14,011	39,675
2010	9,138	25,876
2011	34,832	98,633
2012	35,413	100,278
2013	37,581	106,417
2014	32,048	90,750
2015	26,550	75,181
2016	9,584	27,139
2017	39,889	112,953
