# Supplementary material for: White-tailed deer (Odocoileus virginianus) fawn survival and the influence of landscape characteristics on fawn predation risk in the Southern Appalachian Mountains, USA
Source: PLoS One. 2023 Aug 31;18(8):e0288449. doi: 10.1371/journal.pone.0288449 (PMC10470973; doi:10.1371/journal.pone.0288449)
Supplement: S2 Table — (PDF) [file pone.0288449.s003.pdf]

Chattahoochee National Forest Prescribed Fire

Total area burned – USDA Forest Service data

**Fiscal year**	**Rx burn acres**	**Rx burn ha**
2003	7206	2916
2004	8846	3580
2005	1526	618
2006	8681	3513
2007	4452	1802
2008	7860	3181
2009	9560	3869
2010	11371	4602
2011	6876	2783
2012	7087	2868
2013	16311	6601
2014	22477	9096
2015	13910	5629
2016	16629	6730
