# Supplementary material for: White-tailed deer (Odocoileus virginianus) fawn survival and the influence of landscape characteristics on fawn predation risk in the Southern Appalachian Mountains, USA
Source: PLoS One. 2023 Aug 31;18(8):e0288449. doi: 10.1371/journal.pone.0288449 (PMC10470973; doi:10.1371/journal.pone.0288449)
Supplement: S3 Table — (PDF) [file pone.0288449.s004.pdf]

Cumulative White-tailed Deer Fawn Survival Data

Northern Georgia, USA (2018–2020)

University of Georgia – Warnell School of Forestry and Natural Resources

Fawn ID	capDate	DOB	Entry	Exit	Event	Fate	Cause	Julian DOB	Type	Sex	capMass (kg)	stBirth Mass (kg)	Hoof growth (mm)	Sibling
501	5/28/2018	5/27/2018	2	4	1	natural	nonPred	147	VIT	M	2.50	2.38	1.04	No
503	6/6/2018	6/6/2018	1	71	1	predation	coyote	157	VIT	M	2.18	2.18	0.96	No
505	6/11/2018	6/11/2018	1	5	1	predation	coyote	162	VIT	M	2.24	2.24		No
507	7/17/2018	7/17/2018	1	84	0	recruit		198	VIT	F	2.68	2.68	0.78	Yes
509	7/17/2018	7/17/2018	1	84	0	recruit		198	VIT	F	2.51	2.51	0.7	Yes
513	6/4/2018	6/1/2018	4	8	1	predation	coyote	152	OPP	F	2.41	2.05	1.43	No
515	6/17/2018	6/17/2018	1	24	1	predation	bear	168	VIT	M	2.35	2.35	1.88	Yes
517	6/17/2018	6/17/2018	1	18	1	predation	bobcat	168	VIT	F	1.76	1.76	1.59	Yes
519	6/20/2018	6/18/2018	3	10	1	predation	bear	169	OPP	F	2.41	2.17	1.28	No
521	6/26/2018	6/22/2018	5	45	1	predation	coyote	173	OPP	M	2.32	1.84	1.78	No
527	6/23/2018	6/23/2018	1	84	0	recruit		174	VIT	F	2.68	2.68	1.55	No
530	6/26/2019	6/21/2019	6	8	1	predation	bear	172	OPP	M	2.45	1.85	2.57	Yes
531	6/18/2019	6/18/2019	1	4	1	predation	bobcat	169	VIT	F	1.68	1.68	1.36	Yes
532	6/19/2019	6/19/2019	1	4	1	natural	nonPred	170	OPP	M	2.51	2.51	1.59	No
534	6/15/2019	6/15/2019	1	84	0	recruit		166	VIT	F	2.45	2.45	0.95	No
535	6/22/2019	6/18/2019	5	20	1	natural	nonPred	169	OPP	F	2.13	1.65	2.16	No
536	6/25/2019	6/20/2019	6	32	1	predation	coyote	171	OPP	M	2.23	1.63	2.26	No
537	6/18/2019	6/18/2019	1	4	1	predation	bear	169	VIT	M	2.29	2.29	1.6	Yes
538	6/20/2019	6/13/2019	8	11	1	natural	nonPred	164	OPP	M	2.23	1.39	3.48	No
539	6/23/2019	6/19/2019	5	58	0	drop		170	OPP	M	2.23	1.75	2.17	No
540	6/24/2019	6/22/2019	3	51	0	drop		173	OPP	M	1.91	1.67	0.91	Yes
541	6/26/2019	6/24/2019	3	29	1	predation	coyote	175	OPP	F	2.13	1.89	1.28	No
543	6/23/2019	6/23/2019	1	60	1	predation	coyote	174	VIT	M	2.42	2.42	1.34	No
544	6/26/2019	6/21/2019	6	7	1	predation	bear	172	OPP	M	3.19	2.59	2.54	Yes
545	6/26/2019	6/26/2019	1	30	1	predation	unknown	177	OPP	F	2.01	2.01	1.58	Yes
550	7/1/2019	7/1/2019	1	3	1	predation	bear	182	VIT	M	2.52	2.52	1.47	No
551	7/7/2019	7/5/2019	3	6	1	natural	nonPred	186	OPP	F	2.38	2.14	1.07	No

552	6/26/2019	6/26/2019	1	14	1	predation	coyote	177	OPP	M	2.02	2.02	1.51	Yes
553	7/7/2019	6/30/2019	8	16	1	predation	coyote	181	OPP	M	2.23	1.39	3.55	No
557	7/5/2019	6/30/2019	6	57	0	offline		181	OPP	M	2.23	1.63	2.65	Yes
560	6/28/2019	6/28/2019	1	3	1	predation	unknown	179	OPP	M	2.50	2.5	1.04	No
563	6/27/2019	6/24/2019	4	5	1	natural	nonPred	175	OPP	F	2.13	1.77	1.35	No
581	6/22/2020	6/17/2020	6	9	1	predation	bear	169	OPP	F	3.93	3.33	2.22	No
583	6/23/2020	6/21/2020	3	84	0	recruit		173	OPP	M	3.00	2.76	1	Yes
586	6/27/2020	6/20/2020	8	66	1	predation	bobcat	172	OPP	F	4.11	3.27	1.32	Yes
587	7/10/2020	7/10/2020	1	14	1	predation	coyote	192	VIT	F	2.46	2.46	1.64	Yes
588	6/29/2020	6/27/2020	3	84	0	recruit		179	OPP	M	3.43	3.19	1.11	No
589	6/27/2020	6/20/2020	8	12	1	predation	bobcat	172	OPP	F	4.66	3.82		Yes
590	6/23/2020	6/20/2020	4	5	1	predation	coyote	172	OPP	F	3.42	3.06	1.54	No
591	6/19/2020	6/19/2020	1	34	1	predation	coyote	171	VIT	M	3.43	3.43	1.09	No
592	6/26/2020	6/25/2020	2	25	1	predation	coyote	177	VIT	F	2.23	2.11	1.05	No
593	6/24/2020	6/23/2020	2	84	0	recruit		175	OPP	F	3.94	3.82	0.5	No
595	7/6/2020	7/6/2020	1	8	1	predation	bear	188	VIT	M	2.10	2.1	2.15	Yes
596	6/17/2020	6/14/2020	4	26	0	drop		166	OPP	M	2.18	1.82	1.74	No
597	6/27/2020	6/21/2020	7	63	0	offline		173	OPP	M	4.21	3.49	3.09	No
599	7/4/2020	7/2/2020	3	51	0	offline		184	OPP	F	2.07	1.83	0.88	No
600	6/16/2020	6/14/2020	3	27	1	predation	coyote	166	OPP	M	3.15	2.91	0.88	No
601	7/16/2020	7/15/2020	2	5	1	predation	bobcat	197	VIT	M	2.20	2.08	2.94	Yes
604	6/25/2020	6/23/2020	3	13	1	predation	unknown	175	OPP	F	3.00	2.76	0.91	No
605	7/10/2020	7/10/2020	1	6	1	predation	bear	192	VIT	F	2.47	2.47	1.18	Yes
607	6/25/2020	6/24/2020	2	4	1	natural	nonPred	176	OPP	M	2.64	2.52	0.81	No
608	6/8/2020	6/6/2020	3	56	1	predation	coyote	158	VIT	M	2.64	2.4	1.78	No
609	6/14/2020	6/10/2020	5	36	1	predation	coyote	162	OPP	M	2.45	1.97	2.1	No
611	7/6/2020	7/6/2020	1	16	1	predation	bear	188	VIT	F	1.95	1.95	1.89	Yes
612	6/23/2020	6/20/2020	4	15	1	predation	coyote	172	OPP	F	1.90	1.54	1.39	No
613	6/26/2020	6/24/2020	3	6	1	predation	bobcat	176	OPP	M			1.21	No
614	6/16/2020	6/15/2020	2	84	0	recruit		167	VIT	M	1.77	1.65	2.07	Yes
615a	7/15/2020	7/15/2020	1	43	1	predation	coyote	197	VIT	M	2.11	2.11	2.28	Yes
615b	7/15/2020	7/15/2020	1	2	1	predation	unknown	197	VIT					Yes
616	6/26/2020	6/25/2020	2	4	1	predation	coyote	177	OPP	M	2.96	2.84	0.73	No
617	6/21/2020	6/21/2020	1	11	1	predation	coyote	173	VIT	F	2.69	2.69	2.58	No
618	6/17/2020	6/17/2020	1	4	1	natural	nonPred	169	OPP	M	2.59	2.59	0.24	Yes
619	6/16/2020	6/15/2020	2	84	0	recruit		167	VIT	F	1.68	1.56	1.18	Yes

620	7/16/2020	7/15/2020	2	9	1	predation	coyote	197	VIT	F	2.49	2.37	1.41	Yes
622	6/18/2020	6/18/2020	1	7	1	natural	nonPred	170	VIT	F	2.20	2.2	2.17	Yes
623	6/18/2020	6/18/2020	1	3	1	predation	bear	170	VIT	F	2.35	2.35	1.07	Yes
624	6/17/2020	6/11/2020	7	10	0	drop		163	OPP	M	2.97	2.25	2.97	No
625	7/1/2020	6/26/2020	6	35	1	predation	bobcat	178	OPP	M	3.76	3.16	2.27	No
627	6/26/2020	6/25/2020	2	12	1	predation	coyote	177	OPP	F	3.07	2.95	0.59	No
628	6/11/2020	6/9/2020	3	4	1	predation	bear	161	VIT	M	2.80	2.56	1.08	No
629	6/17/2020	6/15/2020	3	6	1	natural	nonPred	167	OPP	M	2.64	2.4	0.87	Yes
