# Supplementary material for: White-tailed deer (Odocoileus virginianus) fawn survival and the influence of landscape characteristics on fawn predation risk in the Southern Appalachian Mountains, USA
Source: PLoS One. 2023 Aug 31;18(8):e0288449. doi: 10.1371/journal.pone.0288449 (PMC10470973; doi:10.1371/journal.pone.0288449)
Supplement: S4 Table — (PDF) [file pone.0288449.s005.pdf]

Subset of White-tailed Deer Fawn Survival Data with Landscape Characteristics of Fawn Usage Areas

Northern Georgia, USA (2018–2020)

University of Georgia – Warnell School of Forestry and Natural Resources

Fawn ID	Entry	Exit	Event	Frac[a]	SHDI[b]	TRI[c]	Build count	River/ stream (m)	Paved road (m)	Gravel road (m)	Edge[d] (m)	Forest (ha)	Mixed[e] (ha)	Ever[f] (ha)	Deci[g] (ha)	Early succ[h] (ha)	Grass[i] (ha)	Rhodo[j] (ha)
503	1	71	1	1.11	0.94	6.87	0	0.00	0.00	398.08	0.00	14.40	8.55	2.16	3.69	0.00	0.00	0.81
505	1	5	1	1.08	1.05	3.12	4	432.49	119.99	638.72	990.00	11.34	6.75	4.59	0.00	0.00	2.97	0.90
507	1	84	0	1.11	0.45	4.39	0	0.00	0.00	625.10	0.00	14.31	11.97	2.34	0.00	0.00	0.00	7.92
509	1	84	0	1.08	0.43	4.43	0	0.00	0.00	639.74	0.00	14.49	12.24	2.25	0.00	0.00	0.00	7.65
513	4	8	1	1.10	1.03	2.35	0	458.59	0.00	373.08	600.00	13.32	5.94	6.75	0.63	0.90	0.00	3.06
515	1	24	1	1.08	1.05	5.13	0	436.14	0.00	791.10	510.00	13.59	7.11	5.40	1.08	0.63	0.00	2.70
517	1	18	1	1.08	1.05	5.13	0	436.14	0.00	791.10	510.00	13.59	7.11	5.40	1.08	0.63	0.00	2.70
519	3	10	1	1.05	0.87	3.00	1	0.00	315.19	650.34	600.00	14.22	4.41	8.64	1.17	0.00	0.00	4.95
521	5	45	1	1.09	1.09	2.50	10	893.88	0.00	376.43	1080.00	9.27	5.13	0.00	4.14	0.00	4.95	0.99
527	1	84	0	1.08	0.53	3.63	0	0.00	0.00	251.70	0.00	14.22	11.07	3.15	0.00	0.00	0.00	2.52
530	6	8	1	1.10	0.91	7.17	0	0.00	0.00	500.79	0.00	14.40	9.00	2.07	3.33	0.00	0.00	3.69
531	1	4	1	1.10	0.53	3.87	0	0.00	0.00	0.00	0.00	14.49	11.25	3.24	0.00	0.00	0.00	4.95
534	1	84	0	1.12	0.58	4.35	0	0.00	0.00	645.90	0.00	13.31	10.44	2.87	0.00	0.00	0.00	8.46
536	6	32	1	1.10	0.45	4.01	0	526.56	0.00	0.00	0.00	14.04	12.15	0.27	1.62	0.00	0.00	5.13
537	1	4	1	1.11	0.56	3.98	0	0.00	0.00	0.00	0.00	14.04	10.62	3.42	0.00	0.00	0.00	5.31
541	3	29	1	1.13	0.68	3.53	0	469.16	0.00	496.56	0.00	14.22	5.94	8.28	0.00	0.00	0.00	4.14
543	1	60	1	1.09	0.66	6.21	0	453.95	0.00	365.35	300.00	13.86	11.61	1.89	0.36	0.54	0.00	6.21
544	6	7	1	1.10	0.95	7.56	0	0.00	0.00	499.67	0.00	14.31	8.19	1.98	4.14	0.00	0.00	2.97
545	1	30	1	1.07	1.43	3.87	1	574.83	416.99	411.50	1460.00	9.72	5.94	2.34	1.44	1.08	3.51	6.57
550	1	3	1	1.05	0.75	9.80	0	294.98	0.00	357.74	0.00	14.13	2.97	0.99	10.17	0.00	0.00	0.99
552	1	14	1	1.07	1.43	3.87	1	574.83	416.99	411.50	1460.00	9.72	5.94	2.34	1.44	1.08	3.51	6.57
553	8	16	1	1.07	0.96	3.35	0	0.00	0.00	143.77	270.00	13.05	5.76	7.20	0.09	1.26	0.00	7.74
560	1	3	1	1.08	1.24	2.27	7	502.70	148.76	784.97	870.00	9.09	5.22	0.99	2.88	0.00	5.31	0.99
581	6	9	1	1.08	0.50	5.06	0	964.42	0.00	280.90	0.00	14.22	2.88	11.34	0.00	0.00	0.00	4.23
583	3	84	0	1.07	1.08	3.90	6	815.89	0.00	606.54	780.00	10.71	5.31	0.00	5.40	0.00	3.60	3.33
586	8	66	1	1.06	0.48	5.44	0	279.97	330.07	552.56	0.00	14.13	11.52	2.61	0.00	0.00	0.00	11.52
587	1	14	1	1.07	0.57	5.14	0	441.38	0.00	447.51	0.00	14.22	11.70	0.72	1.80	0.00	0.00	5.94
588	3	84	0	1.06	0.63	3.49	0	123.51	0.00	459.68	0.00	14.22	11.25	0.72	2.25	0.00	0.00	0.90

589	8	12	1	1.08	0.45	5.77	0	321.71	376.69	535.73	0.00	14.13	11.79	2.34	0.00	0.00	0.00	10.44
590	4	5	1	1.06	0.74	4.37	0	442.47	0.00	433.38	0.00	14.40	8.91	5.22	0.27	0.00	0.00	5.31
591	1	34	1	1.10	0.51	5.58	0	0.00	0.00	703.50	0.00	14.31	11.34	2.97	0.00	0.00	0.00	8.19
592	2	25	1	1.09	1.06	2.02	0	422.69	0.00	905.84	660.00	13.14	7.29	4.95	0.90	0.90	0.00	3.33
593	2	84	0	1.08	1.08	7.34	5	375.40	0.00	502.68	1290.00	8.01	4.23	3.78	0.00	0.00	6.12	1.17
595	1	8	1	1.10	0.69	4.08	0	0.00	0.00	13.82	0.00	14.22	7.47	0.00	6.75	0.00	0.00	3.24
600	3	27	1	1.07	1.11	3.27	0	0.00	0.00	457.81	450.00	11.79	8.37	1.89	1.53	2.25	0.00	8.46
601	2	5	1	1.06	1.18	4.44	5	740.88	0.00	628.16	1650.00	7.74	5.04	1.35	1.35	0.00	6.39	1.35
604	3	13	1	1.07	0.80	5.45	0	0.00	529.49	310.47	0.00	14.40	8.91	4.86	0.63	0.00	0.00	9.72
605	1	6	1	1.08	0.61	3.14	0	437.52	0.00	451.88	0.00	14.22	11.43	0.72	2.07	0.00	0.00	5.67
608	3	56	1	1.06	0.95	4.61	2	0.00	475.53	174.24	510.00	12.78	5.13	7.65	0.00	0.00	1.62	10.62
609	5	36	1	1.10	0.93	7.35	7	0.00	519.69	620.89	510.00	13.05	5.76	7.29	0.00	0.00	1.35	3.78
611	1	16	1	1.10	0.69	4.23	0	0.00	0.00	30.25	0.00	14.22	7.20	0.00	7.02	0.00	0.00	3.78
612	4	15	1	1.08	0.97	4.53	0	392.81	0.00	831.68	0.00	14.13	5.67	6.75	1.71	0.00	0.00	4.50
613	3	6	1	1.08	0.77	2.06	0	0.00	0.00	441.12	0.00	14.22	9.90	3.42	0.90	0.00	0.00	3.69
614	2	84	0	1.12	0.60	3.00	0	387.38	0.00	775.60	0.00	14.31	4.14	10.17	0.00	0.00	0.00	4.23
615a	1	43	1	1.07	1.24	4.43	3	773.86	0.00	624.61	1380.00	9.54	6.75	1.44	1.35	0.27	4.59	1.98
615b	1	2	1	1.07	1.24	4.43	3	773.86	0.00	624.61	1380.00	9.54	6.75	1.44	1.35	0.27	4.59	1.98
616	2	4	1	1.07	0.79	3.45	0	0.00	531.39	311.26	0.00	14.22	8.82	4.86	0.54	0.00	0.00	9.72
617	1	11	1	1.07	0.72	2.06	0	673.82	0.00	967.25	90.00	14.04	6.12	7.92	0.00	0.09	0.00	3.24
619	2	84	0	1.12	0.60	3.27	0	387.38	0.00	775.60	0.00	14.31	4.14	10.17	0.00	0.00	0.00	4.23
620	2	9	1	1.06	1.18	4.83	5	740.88	0.00	628.16	1650.00	7.74	5.04	1.35	1.35	0.00	6.39	1.35
623	1	3	1	1.06	1.23	3.55	0	0.00	0.00	447.29	870.00	13.05	6.75	2.79	3.51	1.26	0.00	4.05
625	6	35	1	1.12	0.61	3.46	0	100.67	0.00	647.92	0.00	14.13	9.90	4.23	0.00	0.00	0.00	0.72
627	2	12	1	1.11	0.68	3.58	0	334.56	0.00	883.20	0.00	14.22	8.10	6.12	0.00	0.00	0.00	0.72
628	3	4	1	1.09	0.54	2.21	0	327.04	462.43	193.41	0.00	14.40	11.07	3.33	0.00	0.00	0.00	8.82

[a] Frac = mean patch fractal dimension
[b] SHDI = Shannon's Diversity Index
[c] TRI = Terrain Roughness Index
[d] Edge = linear edge between forest type patches and open type patches
[e] Mixed = mixed evergreen and deciduous forest
[f] Ever = evergreen forest
[g] Deci = deciduous forest
[h] Early succ = early successional land cover
[i] Grass = grass lawn and pasture land cover
[j] Rhodo = evergreen understory land cover including *Rhododendron maximum* and *Kalmia latifolia*
